# Cannabinoid CB2 Receptors in Neurodegenerative Proteinopathies: New Insights and Therapeutic Potential

**DOI:** 10.3390/biomedicines10123000

**Published:** 2022-11-22

**Authors:** Barbara Vuic, Tina Milos, Lucija Tudor, Marcela Konjevod, Matea Nikolac Perkovic, Maja Jazvinscak Jembrek, Gordana Nedic Erjavec, Dubravka Svob Strac

**Affiliations:** Division of Molecular Medicine, Rudjer Boskovic Institute, Bijenicka Cesta 54, 10 000 Zagreb, Croatia

**Keywords:** neurodegenerative diseases, proteinopathies, endocannabinoid system, cannabinoid CB2 receptors, neuroinflammation, therapy

## Abstract

Some of the most prevalent neurodegenerative disorders, including Alzheimer’s and Parkinson’s disease, are proteinopathies characterized by the accumulation of specific protein aggregates in the brain. Such misfolded protein aggregates can trigger modulation of the innate and adaptive immune systems and subsequently lead to chronic neuroinflammation that drives the onset and progression of neurodegenerative diseases. Since there is still no effective disease-modifying treatment, new therapeutic targets for neurodegenerative proteinopathies have been sought. The endocannabinoid system, and in particular the cannabinoid CB2 receptors, have been extensively studied, due to their important role in neuroinflammation, especially in microglial cells. Several studies have shown promising effects of CB2 receptor activation on reducing protein aggregation-based pathology as well as on attenuating inflammation and several dementia-related symptoms. In this review, we discuss the available data on the role of CB2 receptors in neuroinflammation and the potential benefits and limitations of specific agonists of these receptors in the therapy of neurodegenerative proteinopathies.

## 1. Introduction

Proteinopathies, characterized by the accumulation and deposition of misfolded and aggregated proteins in various organs [1], have been associated with nearly fifty different diseases [2]. Due to the specific profile of brain tissue structure and function, many human proteinopathies are associated with the central nervous system (CNS). These include some of the most common neurodegenerative disorders, such as Alzheimer’s disease, Parkinson’s disease, Huntington’s disease, lateral amyloid sclerosis, as well as dementia with Lewy bodies, frontotemporal lobar degeneration, prion diseases such as Creutzfeldt–Jacob disease and others [3]. These neurodegenerative proteinopathies affect millions of people worldwide and impose an enormous socioeconomic burden.

Despite extensive scientific research, the complex etiology of these disorders is still unclear, and there is no effective cure, although some treatments may alleviate the symptoms. The hypothesis linking misfolded protein aggregates and neurodegeneration proposed that altered proteins acquire toxic functions or lose their physiological functions and form aggregates due to post-translational protein modifications, loss of protein clearance, or increased protein production, which subsequently lead to neuronal damage and death [4,5]. All neurodegenerative proteinopathies share similarities in their underlying pathological mechanisms; however, their clinical symptoms and prognosis may vary and depend on the affected brain region, the different proteins involved in the aggregate formation, and functional protein variants (isoforms/proteoforms/strains) underlying specific molecular insult mechanisms [1,6]. In addition, there is growing evidence of the involvement of the innate and adaptive immune systems, as well as chronic inflammation, in the pathophysiology of neurodegenerative disease, including protein misfolding, suggesting that potential immunotherapeutic strategies may be useful for the treatment of neurodegenerative proteinopathies [3,7].

The endocannabinoid system (ECS) is a complex biological signaling system present throughout the body that plays an important role in the regulation and homeostasis of numerous physiological processes, including neuro-immune interactions, and has been implicated in the pathophysiology of several neurodegenerative diseases [8,9]. Significant advances in cannabinoid research, as well as the detection of cannabinoid receptors 2 (CB2R) in the brain, have renewed interest in targeting components of ECS as treatment options in CNS disorders [9,10]. In contrast to cannabinoid receptors 1 (CB1R), which are abundantly expressed in most brain regions, CB2R were previously considered to be restricted to peripheral tissues and predominantly expressed by immune cells. Recently, however, these receptors were found to be strongly upregulated in the brain in CNS diseases characterized by neuroinflammatory processes and microglial cell activation [11]. Pharmacological modulation of CB2R has shown positive immunomodulatory and neuroprotective effects in reducing aggregated protein deposition, suggesting the therapeutic potential of natural and synthetic CB2R ligands in neurodegenerative proteinopathies [8,9].

This review will therefore summarize the literature data obtained in previous preclinical and clinical work on the role of ECS and, in particular, CB2R in neuroinflammation, neurodegeneration and neuroprotection, with particular emphasis on their involvement in various neurodegenerative proteinopathies, such as Alzheimer’s disease, Parkinson’s disease, Huntington’s disease and amyotrophic lateral sclerosis, as well as multiple sclerosis. A recent study showed that in multiple sclerosis, which is both an autoimmune and neurodegenerative disease, neuroinflammation triggers the accumulation of toxic protein bassoon in neuronal somata [12]. These findings suggest that multiple sclerosis could also be considered a neurodegenerative proteinopathy, the first with clinically approved cannabinoid therapy (Sativex). Although some findings are promising, further research is needed to fully evaluate the potential benefits, as well as limitations of specific drugs targeting CB2R for the therapy of neurodegenerative proteinopathies.

## 2. Neurodegenerative Proteinopathies

Neurodegenerative proteinopathies is an umbrella term for neurodegenerative disorders characterized by the formation of misfolded protein aggregates that cause cellular toxicity and contribute to cellular proteostatic collapse [13]. According to the pathophysiological hypothesis of neurodegenerative disorders, some proteins change their conformations, consequently gaining neurotoxic activity or losing their normal function by forming small oligomeric or large fibrillar aggregates, leading to neurodegeneration [3]. Neurodegenerative proteinopathies include some of the most common neurodegenerative disorders, such as Alzheimer’s and Parkinson’s disease, as well as Huntington’s disease, multiple sclerosis, amyotrophic lateral sclerosis, etc. The recent findings showing that bassoon proteinopathy drives neurodegeneration in mice and patients with multiple sclerosis are reminiscent of disease pathways in neurodegenerative proteinopathies [12].

The neuronal proteome consists of 10,000 to 20,000 different proteins that, in order to fulfill their biological function, must fold in accordance with the instructions encoded in the amino acid sequence [14]. Therefore, to maintain cellular integrity and health, the process of protein folding and its degradation must be well-regulated [15]. However, their biologically active conformation (the native state) is often marginally stable under normal physiological conditions, and even a small polypeptide of ~100 amino acids can adopt many conformations (~10^30^) under different conditions [14]. It is, therefore, hardly surprising that the process of protein folding is error-prone, leading to misfolded states and off-pathway aggregates [14]. Due to this susceptibility, cells face a continuous stream of misfolded and aggregated proteins (Table 1) and require supportive molecular chaperones called heat shock proteins (Hsp) to refold, degrade, and eliminate them to maintain proteome homeostasis [13].

Under proteotoxic stress conditions induced by reactive oxygen species (ROS), toxins, cell aging, or disease-related gene mutations, proteins can change conformation. When such misfolded proteins escape cellular quality control, they can begin to aggregate into non-native structures, ranging from oligomers and amorphous assemblies to highly ordered amyloid fibrils and plaques [16]. These structures have the potential to disrupt proteostasis and thus impair normal cellular function [15]. Cellular protein homeostasis or proteostasis refers to the integrated activity of cellular mechanisms involved in the regulation of protein synthesis, folding, translocation, assembly/disassembly, and degradation [17]. For example, the heat shock response and the response to unfolded protein involve the transcriptional regulation of various chaperones (e.g., Hsp70 and Hsp90) and non-chaperone proteins such as transcriptional factors, regulators of the cell cycle, as well as signaling receptors and proteins [17]. In addition, during the ageing process or in disorders associated with misfolded proteins, cells can undergo proteostatic collapse or a condition associated with the accumulation of ubiquitinated inclusion bodies [18]. These ubiquitinated inclusion bodies are seen in many neurodegenerative disorders and can directly inhibit or clog the proteasomes [19].

Notably, only single-chain polypeptides can be degraded by proteasomes, requiring the proteins to be partially or fully unfolded [20]. Higher-order amyloid aggregates are particularly resistant to degradation and are extremely thermodynamically stable [21]. This stability contributes to the ability of protein aggregates to propagate in a prion-like manner by changing the normally folded counterparts into pathogenic conformations [21]. Moreover, after the injection of protein aggregates into the brains of normal animals, they can spread to surrounding neurons and neighboring glial cells and induce a new pathology [22]. In addition, misfolding of one protein can cause other susceptible proteins to misfold [22], and therefore aggregates of different misfolded proteins can even be observed in the same patient [23]. Specifically, a particular type of accumulated misfolded proteins can trigger the misfolding of other unrelated proteins that would be properly folded under normal conditions [24]. These mechanisms of interneuronal spreading are currently of great research interest, and some evidence suggests the involvement of activity-dependent secretion by exosomes [25] and/or chaperone-mediated pathways [26].

All of these misfolded and aggregated proteins cause dysfunction and loss of synapses and eventually lead to the death of neurons [13]. Various misfolded proteins are known to cause neurotoxicity, although the exact mechanisms are not yet clear. However, they can act both by toxic gain-of-function and loss-of-normal function [13]. For instance, it has been shown that amyloid β (Aβ), tau and α-synuclein interfere with synaptic signaling [27,28,29]. The mutant tau also disrupts microtubule function and neuronal transport mechanisms, while α-synuclein additionally disrupts mitochondrial protein import [29].

In addition to synaptic dysfunction, one of the most prominent hallmarks of neurodegenerative disorders is cellular distress, characterized by the impairments of mitochondrial function, overproduction of reactive oxygen species, disrupted signaling cascade, and consequent neuroinflammation [13]. These symptoms and the accumulation of misfolded proteins have a bidirectional relationship that is often mutually exacerbating [13]. Aβ, α-synuclein and mutant huntingtin (mHtt) have been shown to induce acute oxidative stress in neurons and reduce the antioxidant capacity of astroglia [30,31,32,33], while conversely, oxidative stress facilitates the aggregation of misfolded proteins and leads to proteostatic breakdown [34,35]. Moreover, both Aβ oligomers and Aβ aggregates stimulate a low level of chronic neuroinflammation by activating microglia and astrocytes [36]. These pro-inflammatory effects of Aβ, in turn, impair microglial and astroglial function as well as their ability to remove Aβ and other misfolded proteins [31,36,37,38]. Finally, the overall process of neuroinflammation caused by misfolded proteins is likely exacerbated by age-related immune system senescence [39,40].

## 3. Endocannabinoid System (ECS)

**Figure 1:**
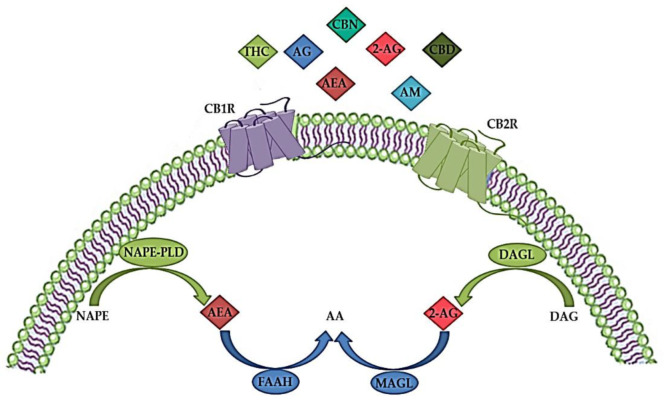
The endocannabinoid system (ECS) consists of endogenous cannabinoids (endocannabinoids) such as anandamide (arachidonoylethanolamide, AEA) and 2-arachidonoylglicerol (2-AG), anabolic enzymes (N-acylphosphatidylethanolamine-hydrolyzing phospholipase D (NAPE-PLD) and 1,2-diacylglycerol lipase (DAGL)), catabolic enzymes (fatty acid amide hydrolase (FAAH) and monoacylglycerol lipase (MAGL)) and cannabinoid receptors 1 (CBR1) and 2 (CB2R). In addition to endocannabinoids, various exogenous natural cannabinoids (phytocannabinoids), such as tetrahydrocannabinol (THC), cannabidiol (CBD) and cannabinol (CBN), as well as synthetic cannabinoids, such as different agonists (AG) or allosteric modulators (AM) act via CBR1 and/or CB2R. AA—arachidonic acid; DAG—diacylglycerol; NAPE—N-acylphosphatidylethanolamine. The image was created using Microsoft PowerPoint 2016.

The ECS plays an important role in both the CNS and peripheral nervous system by modulating the neuronal network function and activity [9]. It is a complex molecular system involved in various biological processes such as maintenance of homeostasis, neurogenesis, neuroprotection, cognition, pain, inflammation, learning and memory, as well as pre- and postnatal development [41,42]. The ECS consists of endogenous cannabinoids (endocannabinoids), cannabinoid receptors (CBR), enzymes and other different proteins important for the transport and metabolism of endocannabinoids (Figure 1) [34,43]. Endocannabinoids are endogenous signaling lipid mediators that activate CBR and mimic the actions of Δ9-tetrahydrocannabinol (THC) [43]. The biological effects of endocannabinoids are mediated by two members of the large family of G-protein-coupled receptors (GPCR); CB1R and CB2R (Figure 1) [29,44].

These receptors are characterized by different signaling mechanisms, tissue distributions and differential expression in neurons and microglia [45]. CB1R are mainly found in the CNS, in regions responsible for motor coordination (cerebellum, substantia nigra, striatum and basal ganglia), cognitive functions (cortex), as well as learning and memory (amygdala and hippocampus), but are also localized in the heart, uterus, testes, liver, gastrointestinal tract, immune cells and adipose tissue [46,47]. In the CNS, CB1R are located in the presynaptic terminals of y-aminobutyric acid (GABA)-ergic, glutamatergic, cholinergic, noradrenergic and serotonergic neurons, and they regulate retrograde suppression of neurotransmission [48]. Their distribution suggests an important role of these receptors in the regulation of cognition, memory and learning processes, movement, and emotions [49], as well as in various neuropsychiatric disorders [48,50]. CB2R are mainly found at the periphery, for instance, in the immune and hematopoietic system, but are activated in the CNS during inflammation, especially in microglia and astrocytes, as well as in oligodendrocytes, neural progenitor cells, and in the endothelium of the blood–brain barrier (BBB) [51], suggesting their immunomodulatory role [49]. Human CB2R has two isoforms; the CB2A isoform is expressed in the testes and brain, while the CB2B isoform is localized in the spleen and leukocytes [52].

Both CB1R and CB2R are seven transmembrane domain receptors coupled to G-proteins (Figure 1). They inhibit adenylyl cyclase, protein kinase A (PKA), and various voltage-gated calcium channels such as N-type, P/Q-type and L-type calcium currents and activate mitogen-activated protein kinases (MAPK) (including extracellular signal-regulated kinases (ERK), c-Jun N-terminal kinases (JNK), and p38 kinases) and inwardly rectifying potassium channels [45,53]. Activation of CB1R blocks the release of various excitatory and inhibitory neurotransmitters and regulates the activity of specific ion channels [54]. In addition, binding to CB1R stimulates signaling pathways such as phosphoinositide 3-kinase (PI3K)/Akt, MAPK and Nrf2 cascades involved in antioxidative defense and survival and activates N-methyl-D-aspartate (NMDA) receptors, Ca^2+^ signaling cascades and influx, thereby regulating glutamatergic signaling [55]. CB2R signaling also suppresses adenylyl cyclase, lowers cAMP levels, and decreases PKA activity [55]. However, cAMP synthesis and activation of Akt and ERK signaling pathways are stimulated by CB2R signaling, probably by regulating different adenylyl cyclase isozymes [54]. The protein kinase C (PKC) pathway, Janus kinase (JAK)/signal transducer and activator of transcription 1 (STAT1) pathway are some of the signaling pathways through which microglial activation is suppressed by CB2R stimulation [56,57].

CBR activation is mediated by the two most common endogenous endocannabinoid ligands, anandamide (arachidonoylethanolamide, AEA) and 2-arachidonoylglicerol (2AG), both of which are derivates of n-6 polyunsaturated (PUFA) arachidonic acid (Figure 1). AEA has high partial agonist affinity to CB1R but low efficacy at CB1R and even lower efficacy at CB2R, whereas 2-AG has low to moderate full agonist affinity to these two receptors but is fully effective [34,58,59]. In addition, AEA is a full agonist at transient receptor potential cation channel subfamily V member 1 (TRPV1), also known as vanilloid receptor 1 (VR1), and at nuclear peroxisome proliferator-activated receptor (PPAR), while 2-AG binds to specific GABA receptor A subtypes in neuronal cells [45,60].

High levels of AEA have been found in different brain regions, such as the hippocampus, thalamus, cerebral cortex, and cerebellum, while lower levels of AEA have been detected in the periphery, including human blood and cerebrospinal fluid [61,62]. On the other hand, 2-AG is present in high concentrations in the brain stem, hippocampus, and striatum [61]. Although AEA and 2-AG have different receptor affinities, synthesis, transport and inactivation pathways, both are produced in response to high intracellular Ca^2+^ concentrations [63,64]. As shown in Figure 1, biosynthesis of AEA from N-arachidonoyl phosphatidylethanolamine by multiple pathways is initiated by a postsynaptic depolarization and an increase in Ca^2+^ ions that activates N-acylphosphatidylethanolamine-hydrolyzing phospholipase D (NAPE-PLD) and diacylglycerol (DAG) lipase [65]. 2-AG is synthesized from arachidonic acid-containing DAG by the action of DAG lipase (Figure 1). DAG lipase alpha is important for the synaptic production of 2-AG in the adult brain, while DAG lipase beta is responsible for the microglial production of 2-AG [66]. Studies have shown that disrupted synaptic localization of DAG lipase alpha is associated with CNS disorders [67].

Due to their uncharged hydrophobic nature, endocannabinoids cannot diffuse easily like other neurotransmitters. There are three different models for the transport of AEA once it is released into the intracellular space [68]: simple diffusion facilitated by the concentration gradient [69], transport by protein carriers, and endocytosis [70]. It is believed that 2-AG has a similar transport pathway, but it is not yet well described [71]. Endocannabinoids taken up by cells can be degraded by two different pathways, hydrolysis and oxidation [68]. Enzymes that are involved in the hydrolysis pathway include fatty acid amide hydrolase (FAAH) for AEA and monoacylglycerol lipase (MAGL) for 2-AG (Figure 1) [66,68,72]. The oxidation of both AEA and 2-AG involves cyclooxygenase (COX) and lipoxygenase (LOX) [68].

ECS dysfunction and its alterations in the CNS are involved in the pathophysiology of neurodegenerative diseases such as Alzheimer’s disease [73], Parkinson’s disease [74], Huntington’s disease [75], multiple sclerosis [76] and amyotrophic lateral sclerosis [48]. In addition, ECS dysregulation has been found in patients with schizophrenia [77], anxiety disorders [78] and major depressive disorder (MDD) [79]. Neuroimmune and neurooxidative pathways are involved in neurocognitive impairments and various behavioral symptoms, as observed in several neuropsychiatric disorders [79,80,81]. Therefore, due to their neuroprotective and neuroinflammatory roles in the CNS, pharmacological modulation of different components of the ECS may have therapeutic potential in various CNS disorders [82].

## 4. Modulation of Cannabinoid Receptor 2 (CB2R)

Marijuana or cannabis (*Cannabis sativa*) contains about 500 compounds, of which at least 100 are classified as phytocannabinoids with different chemical structures and pharmacological properties, the most abundant natural cannabinoids being THC, cannabidiol (CBD) and cannabinol (CBN) [83]. CBR1 and CRBR2 were first studied as targets of THC in the human brain [84]. THC interacts with both CBR, as an agonist at CB1R and as a weak antagonist at CB2R [85], but also possibly by inhibiting COX enzymes and as an inducer of COX-2 with prolonged exposure [86]. In addition, another natural cannabinoid CBD, which has a low affinity for CBR, may be a CB2R inverse agonist with anti-inflammatory effects [87]. Subsequently, the identification of CBR in the brain suggested the presence of endogenous ligands, and the most studied and characterized endocannabinoids are AEA and 2-AG, which have an affinity for both CBR [84,88]. It was found that 2-AG acts as a full agonist, while AEA acts as a weak partial agonist for both CB1R and CB2R [85].

CP 55,940 was the first synthetic cannabinoid analog to be synthesized, followed by several others. Although many synthetic cannabinoids are known to have an affinity for both CB1R and CB2R, they, like natural cannabinoids, can also interact with non-CBR, such as vanilloid or serotonergic receptors [89]. The most common group of synthetic cannabinoids is JWH, where JWH-018 has potent pharmacological activity and can be easily synthesized and used to synthesize other synthetic cannabinoids with different properties and affinities for CBR [90]. Besides JWH, other common groups of synthetic cannabinoids are HU and CP groups. While HU are classic cannabinoids, CP are cannabimimetics originally developed by Pfizer in the 1970s [90]. Some of the most extensively studied selective CB1 or mixed CB1/CB2 agonists are WIN 55,212-2, HU 210, ACEA, and JWH-018 [91]. In addition, several synthetic selective CB2R agonists, such as GSK554418A, GW833972A, GW842166X, HU-308, GW405833, JWH-015, JWH-133, A-836339, AM1241, AM630, NESS400, etc., have been reported in the literature and some (Cannabinir, GW842166, Tedalinab, GRC10693, S-7774698, LY2828360, KHK6188, Lenabasum) are under investigation at various stages of clinical development [85,89].

In humans, CB2R are encoded by the cannabinoid receptor 2 (*CNR2*) gene, which is located on chromosome 1p36 and consists of 360 amino acids [92]. The CB2R share 44% total amino acid homology and 68% homology in the transmembrane domains with the CB1R [92]. The CB2R were cloned in 1993, and these receptors were previously thought to be absent from the brain, as they were only detectable in the periphery [51,93,94].

In contrast to CB1R, which are mainly found in the CNS, particularly in presynaptic neurons at central and peripheral nerve terminals, where they inhibit neurotransmitter release [95], CB2R predominate in cells and tissues involved in the immune response, such as the spleen, thymus and blood-derived monocytes [51,96], and modulate interleukin release and cell migration.

Until recently, the significant increase of CB2R in the CNS was thought to occur specifically in activated microglial cells under inflammatory conditions but was not measurable under physiological conditions or in other brain cell types [97]. However, using methods such as immunostaining, in situ hybridization, and gene expression analysis, CB2R has been shown to be present throughout different brain regions [98,99,100,101], including the striatum, amygdala, hippocampus, cortex and ventral tegmental area [102], in neural progenitor cells, neurons, as well as glial and endothelial cells [103,104,105,106]. In neurons, CB2Rs appear to be mainly distributed in postsynaptic somatodendritic regions, and their activation inhibits neuronal excitability through membrane hyperpolarization [97,98,101]. Novel detection techniques allowed more precise detection of low CB2R mRNA levels, specifically in astrocytes, dopaminergic, glutamatergic and GABAergic neurons, but not in resting microglia [11,107,108,109].

Although there are numerous studies on the regulation of CB1R, knowledge of the physiological and pathological role of CB2R is limited. Activation of CB2R leads to the inhibition of neuroinflammatory signaling pathways, as well as a return from the pro-inflammatory state of microglia to normal anti-inflammatory function [85]. Thus, in in vitro experiments, AEA has been shown to act via the MAPK signaling pathway within the CNS immune system to reduce the magnitude of the inflammatory response, as well as to limit neurodegenerative immune responses [110]. Moreover, AEA was found to reduce lipopolysaccharide-induced neuroinflammation in primary rat microglial cultures [111]. Even though AEA can activate CB1R, CB2R and other receptors of the ECS, the anti-inflammatory actions appear to be mediated by CB2R [112]. Therefore, AEA may have a potential therapeutic effect on microglial-derived neuroinflammation and regulate many aspects of the inflammatory response in the brain. However, since CB2R ligands exert neuroprotection without psychotropic effects (strong mood alterations, anxiety, acute psychosis, cognitive and motor impairments), usually seen with CB1R agonists [95], new and selective CB2R ligands may be promising and safe drugs for the treatment of various neuroinflammatory disorders [111]. Nevertheless, CB2R agonists also have disadvantages, such as immune suppression during chronic use, or pro-inflammatory actions [111].

Only a few synthetic CB2R agonists have reached clinical trials, despite increasing reports of selective CB2R ligands and high expectations with these ECS targets [113]. Some of them, such as GW842166X, CP55940, S-777469 and JTE-907, have already completed Phase II trials in various pain disorders; however, none of them have been assessed for neurodegenerative or neuroinflammatory disorders in humans [111]. Recently, new CB2R ligands have been characterized for their potential neuroprotective effects and the most prominent among them, the inverse agonist of CB2R SMM-189 seems to achieve neuroprotection by modulating microglial activation in a mouse model of mild traumatic brain injury [114]. Specifically, SMM-189 reduces some pro-inflammatory markers, indicating decreased infiltration of peripheral macrophages and other immune cells involved in neurodegeneration [115].

Recently, new strategies targeting CB2R for neurodegenerative and neuroinflammatory disorders have emerged when 4′-O-methylhokiol, the main bioactive component of Magnolia grandiflora L. that acts as both CB2R modulator and COX-2 substrate-specific inhibitor, has shown beneficial effects in animal models of neurodegeneration [116]. Moreover, targeting CB2R homo- and heterodimers needs to be further investigated [111]. While homobivalent and heterobivalent CB1R ligands have been previously designed and described in the literature [117], the first structurally bivalent CB2R compounds were designed and synthesized in 2014; however, with lower activity and selectivity compared to their monomeric counterparts [118]. Whereas monomeric compound is a selective CB2R agonists, bivalent compounds are weak antagonists/inverse agonists at CB1R and CB2R [118].

Another therapeutic possibility is the use of ligand-biased signaling [119]. For example, 2-AG is a very potent activator of the ERK1/2-MAPK signaling pathway at low concentrations, although higher concentrations are required to inhibit the adenylyl cyclase and calcium pathways [120]. In the future, CB2R allosteric modulators may offer new therapeutic approaches due to their potential to fine-tune receptor responses while minimizing the side effects [111]. Currently, the allosteric modulation specific to the CB2R signaling is still evaluated [121], while CB2R positive and negative allosteric modulators remain to be discovered.

## 5. Role of CB2R in Various CNS Disorders

Neurodegenerative disorders, such as Alzheimer’s disease, Parkinson’s disease, Huntington’s disease, multiple sclerosis and amyotrophic lateral sclerosis, are characterized by a progressive loss of specific neurons in various brain regions, which leads to different symptomatic and clinical outcomes [122,123]. Since there is no cure for these diseases, therapeutic approaches mostly consist of partial symptomatic relief, which do not halt the progression of the disease. The main hallmarks of neurodegenerative diseases are neuroinflammation, oxidative stress, abnormal protein accumulation and excitotoxicity [124]. Studies have shown that pharmacological modulation of endocannabinoid signaling can modulate these neurodegeneration traits and cause alleviation of symptoms and disease progression [125]. Targeting different components of ECS, therefore, brings new aspects to understanding mechanisms underlying different CNS disorders to provide novel, more effective therapies.

The role of CB1R in behavior, emotions, learning and memory, addiction and various other CNS disorders has been widely studied [49,126]. Previous studies have shown that dysregulation of CB1R in different CNS regions is involved in the pathophysiology of schizophrenia, MDD and anxiety disorders [127,128,129]. Therefore, normalizing the CB1R activity may have beneficial effects in treating these disorders. However, obtained findings demonstrated that CB1R pharmacological targeting induces serious side effects, such as depression, psychosis, panic attacks, anxiety and even suicidal ideation [130,131]. Hence, there is an emerging need to study new therapeutic targets with minimal adverse effects [130,131]. Recent studies suggested that targeting CB2R in CNS is effective and safe and may open a new possibility for the modulation of ECS.

Compared to CB1R, CB2R have lower expression levels in the brain under normal physiological conditions, but their enhanced levels were observed in neurodegenerative and neuropsychiatric disorders [101,105,132]. Recent findings suggested that B2R modulates the behavioral effects in the CNS [9], including mood and emotional behavior. Evidence suggests CB2R plays a role in food intake, body weight control and eating disorders [133,134,135], depression and anxiety [101,105,134], drug addiction [136], psychosis and schizophrenia-like behavior [137,138,139] and synaptic plasticity underlying cognitive functions [135,138].

Elevated CB2R expression levels have been reported in several pathological conditions, such as neurological pain [136,140], stroke [137], traumatic brain injury [140,141], addiction [142,143] and neurodegenerative diseases, including multiple sclerosis [144,145]. CB2R anti-inflammatory action has been found in animal studies and in experiments using cell cultures [141,146]. The activation of CB2R decreases neuroinflammation, partly by mediating the transition of microglial phenotype from a predominantly neurotoxic ‘’M1” to a neuroprotective ‘’M2” [147], suggesting an important role of CB2R in restoring homeostasis [85]. Therefore, due to CBR2 inducible nature during inflammation, ligands that activate or inhibit their activity could be used for potential therapeutic purposes in various CNS disorders whose pathogenesis involves neuroinflammatory processes [97].

## 6. Role of CB2R in Neuroinflammation and Neurodegeneration

A strong relationship between neuroinflammation and neurodegeneration has been reported in the early stages of neurodegenerative disorders, such as Alzheimer’s disease, frontotemporal dementia, Parkinson’s disease, amyotrophic lateral sclerosis and Huntington’s disease. This link is also strong in primarily inflammatory diseases such as multiple sclerosis and human immunodeficiency virus (HIV)-associated dementia associated with intense and chronic inflammation of myelin sheets and HIV infection of microglia, respectively, consequently leading to neuronal damage [123]. Moreover, the neuroinflammatory condition is characteristic of other psychiatric disorders and neurological diseases such as epilepsy, and traumatic brain injury, where it mediates secondary neurodegeneration [148,149].

**Figure 2:**
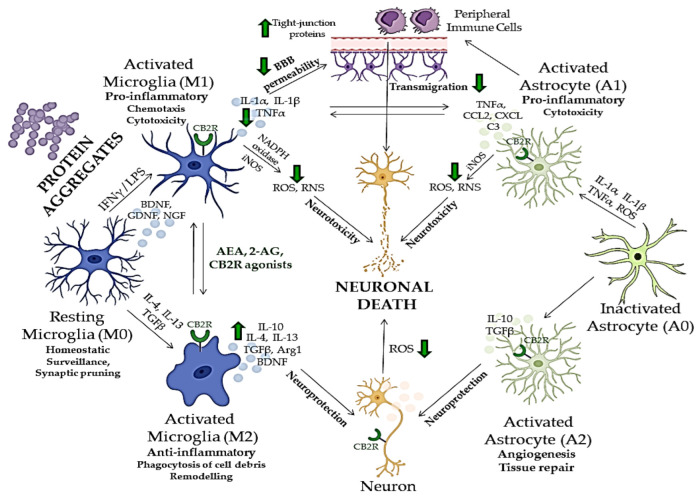
The cross-talk between neuroinflammation and neurodegeneration and neuroprotective effects of compounds acting via CB2R. Green arrows indicate the effects of endocannabinoids or CB2R agonists on different mediators of neuroinflammation and neurodegeneration. BBB—blood–brain barrier; CB2R—cannabinoid receptor 2; ROS—reactive oxygen species; RNS—reactive nitrogen species; iNOS—inducible nitric oxide synthase; IFNγ—interferon gamma, LPS—lipopolysaccharide; IL—interleukin; TGFβ—tumor growth factor beta; TNFα—tumor necrosis factor alpha; BDNF—brain-derived neurotrophic factor; NGF—neuron growth factor; GNDF—glial cell-derived neurotrophic factor; NADPH—nicotinamide adenine dinucleotide phosphate; Arg1—arginase 1; CCL2—C-C motif chemokine ligand 2; CXCL—C-X-C motif chemokine ligand; C3—complement C3. The image was created using Microsoft PowerPoint 2016.

Many aspects of neuroinflammation and neurodegeneration cross-talk remain unclear; however, recent studies showed that glial cells, especially microglia, which act as the brain’s immune cells, could be crucial mediators of neurodegeneration, together with peripheral monocytes which cross BBB under CNS pathological conditions (Figure 2) [150,151,152]. ECS has been, therefore, extensively investigated in relation to neurodegenerative and neuroinflammatory mechanisms of CNS disorders, and potentially novel treatment strategies [153]. Although there are major differences in the etiology, physiology and clinical picture of various neurodegenerative proteinopathies, aggregation and accumulation of defected and misfolded proteins such as Aβ, hyperphosphorylated tau [154], α-synuclein [155], mutated superoxidase dismutase 1 (mSOD1) [156] and huntingtin [157], are shared aspects of these disorders and all represent activation stimulus to circulating microglia [158].

Glial cells play a central role in inducing and maintaining neuronal synaptic plasticity and represent the first line of defense against neuroinflammation [141,159]. Microglia typically occur in three states, distinguished by their receptor expression profile, morphology, and biological functions [11]. Inactivated microglia (M0) are characteristic of homeostatic, non-pathological conditions. Their role is scanning the environment for potential infectious components [160] and regulating the growth and protrusion of dendritic spines [161]. In response to various CNS insults [162], microglia transit from an anti-inflammatory to a reactive pro-inflammatory phenotype (M1), which exhibits cytotoxic and phagocytic activity to eliminate damaged neurons and cellular debris [163]. The activation of resting microglia, as an answer to a threat, leads to microglial polarization, which results in exacerbated neuroinflammation, excitotoxicity and oxidative stress [150]. M1 microglia is characterized by the production and secretion of ROS and reactive nitrogen species (RNS), inflammatory cytokines such as tumor necrosis factor-α (TNF-α), interleukin 1β (IL-1β), IL-6 or IL-12 and recruitment of other immune cells [164,165]. After the threat is gone, M1 microglia switches to an alternative activation state (M2) in which it produces anti-inflammatory and neuroprotective factors, such as IL-10, tumor growth factor-β (TGF-β) and brain-derived neurotrophic factor (BDNF) to dampen further inflammation and induce healing process [165]. However, chronic activation of inflammatory signaling pathways and continuous release of inflammatory cytokines and chemokines by reactive microglia can subsequently result in damage to neurons [166,167], which underlies the pathogenesis of various neurodegenerative disorders (Figure 2) [85].

CBR expression changes over time in the brain and at the periphery, depending on the different stages of neurodegeneration [168]. During neurodegeneration, CB1R-expressing neurons show a progressive loss. For instance, in the early stages of Alzheimer’s disease, the activity of CB1R is increased in the hippocampus, whereas in its advanced stages, decreased CB1R activity has been observed [168]. On the other hand, in a healthy brain, CB2R expression is modest, but it rises in activated astrocytes and microglia [168]. In particular, activated microglia show both increased CB2R expression and higher endocannabinoid synthesis [169]. Upregulated endocannabinoid signaling alleviates microglial over-activation, inhibits pro-inflammatory cytokine release, reduces microglial overactivity, and decreases phagocytic capability [92,170]. One of the main mechanisms by which CB2R might counteract neuroinflammation and attenuate neurodegeneration is changing the microglial polarization, namely shifting it to protective and anti-inflammatory (M0 and M2) states (Figure 2) [169]. For instance, CB2R activation by AEA through activation of ERK1/2 and JNK boosted the expression of anti-inflammatory cytokines (IL-10), characteristic for M2 microglia, and decreased the expression of M1 characteristic markers, while CB2R inhibition inhibited this effect [171]. Additionally, the polarization to the M2 state is disrupted by *CB2R* deletion in microglial cells from *CB2R* knockout mice [169].

Besides microglia, neuronal homeostasis could be maintained by CB2R activation in neurons, leading to a reduction of oxidative damage by influencing the expression of neuronal nitric oxide synthase (NOS), excitotoxicity and apoptosis [172,173]. In astrocytes, which express both CB1R and CB2R, ECS activation leads to the simultaneous production of anti-inflammatory factors and inhibition of pro-inflammatory cytokines, and to lower inducible NOS (iNOS) expression and decreased release of neurotoxic factors [92,174,175]. Additionally, it has been demonstrated that CB2R activation in brain microvascular endothelial cells reduces the tight junction protein expression and BBB permeability after traumatic brain injury, which prevents peripheral immune cells from migrating further into the CNS [176].

Nevertheless, it is reasonable to consider microglia as one of the central factors underlying neurodegenerative pathology, both in a protective and toxic manner [177]. In neurodegenerative proteinopathies, the primary role of activated microglia is the clearance of misfolded proteins and damaged cells, which is followed by a healing phase and neuroprotection. However, the progressive nature of these diseases and the ongoing production of misfolded proteins, continuously activate the cytotoxic state of microglia, which leads to the overly activated inflammatory response and diminished healing possibilities, thereby worsening the clinical picture [177]. ECS and CB2R have been shown to regulate the neuroinflammation in neurodegenerative disorders, mostly in microglia, but also through neuronal and astroglial cells and their cross-talk (Figure 2) [58,168]. Additionally, numerous research studies have shown that both endogenous and exogenous cannabinoids lower the microglial over-activation and effectively ameliorate the neurotoxic effects and neurodegeneration in various neuropsychiatric disorders (Figure 2) [9].

## 7. Role of CB2R in Neuroprotection

Due to their wide spectrum of actions in the CNS and at the periphery [178], cannabinoids might have a potential neuroprotective role in various neurodegenerative disorders [168]. Moreover, endocannabinoid signaling has been involved in many processes which underlie the development of CNS disorders, including oxidative stress, neurotoxicity, neuroinflammation, mitochondrial dysfunction and protein misfolding [168] and therefore might represent a promising target for neuroprotective treatments. CBR might influence neurodegeneration by affecting either neuroprotection or excitotoxicity [168]. CB2R are involved in several biological processes, including differentiation, proliferation and survival of neuronal cells [179], as well as apoptosis induction of encephalitogenic T-cells, leukocyte interference and their adhesion to the endothelium, which are important in the regulation of neurotoxicity and reduction of inflammatory-related impairments of neurons, glial cells and myelin [178].

Several studies focused on the neuroprotective effects of CBR [179]. CB2R may play an important role in neuroprotection by restraining inflammation that leads to neurodegeneration and the development of neurodegenerative disorders [168]. In the activated astrocytes and microglia, CB2R are involved in neuroprotection through the inhibition of chemokine production in astrocytes and the reduction of neurotoxic factors produced by microglia (Figure 2) [180]. While CB2R protect the brain against neuroinflammation by controlling inflammatory processes and the release of cytokines, CB1R are involved in the protection against neuronal death induced by the stimulation of excitatory receptors and calcium release [168]. In addition, CB2R are located on somatodendritic areas and are, therefore, involved in reducing neuronal excitability [97].

Pharmacological modulation of CB2R demonstrated protective actions against anxiety, depression, schizophrenia, autism spectrum disorder, Alzheimer’s disease, Parkinson’s disease, Huntington’s disease, multiple sclerosis, amyotrophic lateral sclerosis, epilepsy and traumatic brain injury [9]. Therapeutic approaches that target CB2R might help alleviate various neuropsychiatric and neurodegenerative disorders by avoiding typical CB1R—mediated symptoms, such as depression and anxiety [181]. However, some studies have shown that in Parkinson’s disease, CB2R agonists lack neuroprotective effects, which were achieved only with antioxidant cannabinoids [179].

## 8. CB2R in Alzheimer’s Disease

Alzheimer’s disease is a leading cause of dementia in the elderly population, contributing to 60–70% of all dementia cases. Due to population aging and the increase in dementia prevalence by age, it is estimated that the number of people suffering from dementia will rise dramatically and reach 152.8 million cases by 2050 [182]. The main neuropathological hallmarks of Alzheimer’s disease are neuritic plaques, the extracellular deposits of Aβ protein, as well as neurofibrillary tangles, the intracellular aggregates of hyperphosphorylated tau protein. Aβ-plaques and neurofibrillary tangles, accompanied by neuroinflammation, oxidative stress, excitotoxicity, etc., lead to neurodegeneration and cognitive dysfunction. The accumulation of extracellular Aβ-plaques and intracellular neurofibrillary tangles damages neurons and leads to neuronal death. Microglia have a crucial role in protecting neurons by detecting and phagocyting Aβ-plaques. Another ability of microglia is to form a barrier around Aβ-plaques, thus preventing their neurotoxic effect on neurons [183]. Microglia-mediated protection also refers to their phagocytosis of extracellular neurofibrillary tangles during the late stages of Alzheimer’s disease [184]. Apart from these modes of action, microglia stimulate the secretion of pro-inflammatory chemokines and cytokines, nitric oxide and free radicals in contact with the Aβ-plaques, thus leading to neuroinflammation [185].

The interest in CB2R as a potential therapeutic target in Alzheimer’s disease arose from the fact that these receptors modulate inflammatory processes and protect the brain by regulating the migration and infiltration of microglia into brain areas, which are affected by neuroinflammation and degeneration [170,179,186]. Increased levels of CB2R have been detected in astrocytes and microglia surrounding neuritic plaques [145,187,188]. CB2R activation induces an anti-inflammatory phenotype of microglia and reduces microglial migration via the induction of MAPK-phosphatase (MKP) [141]. CB1R and CB2R were both localized in Aβ-plaques, and CB2R-specific staining was detected in tangle-like neurons and dystrophic neurites [187,188]. However, CB2R expression was not associated with cognitive impairment measured with MMSE, even though it was suggested to correlate with Aβ42 levels and senile plaque scores [145].

The involvement of CB2R in Alzheimer’s disease pathology has been explored by in vitro and in vivo approaches. In vivo studies were mostly based on different genetic mouse models of Alzheimer’s disease, which reflect the main neuropathological hallmarks of the disease. Different studies using animal models support the association between CB2R and pathological changes in Alzheimer’s disease. For instance, mice lacking CB2R were found to have tau neuropathology, impairment of hippocampus-dependent memory, and mitochondria dysfunction [189]. CB2R deletion was also associated with increased Aβ42 and plaque deposition in a mouse model overexpressing human amyloid precursor protein (APP) [190].

On the other hand, CB2R activation by the agonist JWH-015 induced the clearance of Aβ in frozen human tissue samples and human macrophage cell lines [191]. Long-term treatment of transgenic APP/PS1 mice with JCW-015 decreased microglial phenotype conversion and restored dendritic complexity in the cortex [192]. In addition, the JCW-015 treatment resulted in the normalization of the cortex-dependent memory deficit evaluated with the novel object recognition test but no effect on the hippocampus-dependent spatial cognitive dysfunction estimated by the Morris water maze test [192]. Aso and colleagues described cognitive improvement in APP/PS1 transgenic mice treated with the CB2R agonist JWH-133, supporting the role of CB2R in cognitive functions [193]. This study associated cognitive improvement with reduced microglia reactivity and expression of pro-inflammatory cytokines [193].

Chronic administration of JWH-133 in Tg APP 2576 mice resulted in reduced TNF-α levels and lower activation of microglia, while the reduction in the Aβ-plaque load was associated with improvement in cognitive performance [194]. Another CB2R agonist, MDA7, suppressed neuroinflammation and triggered clearance of Aβ-plaques in the APP/PS1 mice model of Alzheimer’s disease [195]. Additionally, treatment with MDA7 was related to better performance in the Morris water maze task, suggesting improvement in spatial learning and memory [195]. Treatment with MDA7 promoted the clearance of Aβ-plaques, restored synaptic plasticity, cognition, and memory, reduced expression of specific microglia markers and decreased the secretion of pro-inflammatory cytokines [196]. However, CB2R deletion was also associated with the improvement of cognitive and learning abilities [197]. In APP/PS1*CB2^−/−^ mice, cognitive improvement was accompanied by attenuated neuronal loss, decreased plaque load and elevated expression of Aβ-degrading enzymes, suggesting a beneficial role of CB2R deficiency in transgenic mouse models characterized with APP overexpression [197]. In line with these results, tau overexpression was linked to increased CB2R expression in the hippocampus of transgenic mice with the hTAUP301S protein overexpression [198]. These findings suggest that in contrast to the induction of CB2R expression in microglia, CB2R overexpression in neurons accelerates the neurodegenerative process [198].

Most of the above-mentioned studies propose a beneficial role of CB2R activation in the pathogenesis of Alzheimer’s disease. The findings support the role of CB2R in modulating neuroinflammation, which could be the main mechanism of CB2R improvement of cognitive functions in Alzheimer’s disease, and emphasize the relevance of timing of CB2R activation (pre-symptomatic vs. early symptomatic vs. late symptomatic phase). Komorowska-Müller and colleagues pointed out the importance of including the time-dependent CB2R expression profile in future studies to clarify the role of CB2R in microglial activation at different stages of Alzheimer’s disease [11].

## 9. CB2R in Parkinson’s Disease

Parkinson’s disease is the second most common (affecting 1% of the elderly population) neurodegenerative disorder, characterized by a progressive loss and neurodegeneration of dopaminergic neurons, primarily in the substantia nigra [102,199]. Typical symptoms of this disease include impairments in motor function, tremors, rigidity and postural instability. The pathogenesis of Parkinson’s disease involves the formation of Lewy bodies, as well as impairments in several cellular processes, including mitochondrial dysfunction, oxidative stress, protein misfolding, calcium dysregulation, as well as neuroinflammation, which is reflected through microglia activation and cytokine level increase [9,102,200,201]. Dopamine replacement therapy using levodopa is the most commonly used treatment for Parkinson’s disease due to its efficiency in treating typical symptoms, such as bradykinesia and rigidity. However, there is no effective therapy that can delay neurodegeneration and slow down the progression of the disease [199,202].

Recently, due to the observed neuroprotective effects of cannabinoids, the role of CBR, especially CB2R, in Parkinson’s disease has been extensively studied [102,202]. In addition to other brain regions, CB2R are located in dopaminergic neurons of the nigrostriatal pathway, and therefore activation of these CB2 receptors might affect the progression of Parkinson’s disease through regulation of neuronal signaling and function, neurotransmission and neuroinflammation [102].

In animal models of Parkinson’s disease, CB2R activation increased the capacity of antioxidant enzymes, suggesting their role in reducing oxidative stress, excitotoxicity and neuroinflammation, which may subsequently slow down the progression of the disease [102]. CB2 receptor activation prevented nigrostriatal neurodegeneration, inhibited the release of pro-inflammatory cytokines and gliosis and reduced the number of activated astrocytes and microglia [203]. For example, several studies have shown that CB2R agonists (JWH-133, HU-308, JWH-015) exert neuroprotective effects by decreasing inflammation and microglia activity, inhibiting the release of pro-inflammatory cytokines and promoting the release of anti-inflammatory cytokines, as well as increasing glutamate uptake [102,168]. Moreover, CB2R agonist AM-1241 regenerated dopaminergic neurons in substantia nigra in animals with drug-induced Parkinsonian symptoms [9], whereas a natural CB2R agonist, β-caryophyllene, in a rat model of Parkinson’s disease, enhanced the activity of antioxidant enzymes, superoxide dismutase and catalase, and thus attenuated oxidative stress [203]. On the other hand, a lack of CB2R lead to increased activation of microglial cells and degeneration of dopaminergic neurons.

Similarly, clinical studies have also reported increased CB2R expression in different brain cells, including microglia, while lower CB2R levels in dopaminergic neurons were observed in the putamen and substantia nigra of patients with Parkinson’s disease in comparison to control subjects [9,199,204]. Another study found that CB2R gene expression was increased in the substantia nigra but decreased in the putamen of patients with Parkinson’s disease [205]. However, in this study, the altered CB2R expression has been observed only in astrocytes but not in microglia or dopaminergic neurons [205].

Therefore, future studies are necessary to elucidate the mechanisms of the involvement of ECS and CB2R in Parkinson’s disease. Due to their neuroprotective and anti-inflammatory role observed in preclinical and clinical studies of Parkinson’s disease, ECS and CB2 receptors could represent potential therapeutic targets for alleviating symptoms, as well as for slowing the progression of the disease [9,200].

## 10. CB2R in Huntington’s Disease

Huntington’s disease is a progressive neurodegenerative disorder caused by a lethal autosomal dominant mutation in the gene coding for huntingtin (*HTT*). The expansion of CAG repeats in *HTT* exon 1 results in an elongated polyglutamine sequence within the huntingtin N-terminal domain [206]. In healthy subjects, *HTT* has up to 35 CAG repeats, but individuals with Huntington’s disease have more than 37 CAG repeats [207]. Deposition of N-terminal fragments of huntingtin results in intracellular protein aggregates or inclusion bodies [208]. However, the contribution of soluble and intracellular protein aggregates derived from mutant huntingtin (mHtt) to the pathogenesis of Huntington’s disease is still unclear.

The average age of onset for Huntington’s disease is around 40 years of age [209], and the prevalence is around 5.70 per 100,000 inhabitants worldwide, with a mean incidence of around 0.38 per 100,000 per year [210]. Huntington’s disease is characterized by motor impairment, cognitive dysfunction and mental health difficulties [211]. The main neuropathological hallmarks of Huntington’s disease are the progressive degeneration of neurons in the striatum and the cortex, neuroinflammation and progressive accumulation of reactive microglia [212]. Elevated levels of several inflammatory cytokines, including IL-1β, IL-6, IL-8, and TNF-α, detected in CNS and plasma from patients with Huntington’s disease, indicate the importance of neuroinflammation in disease pathogenesis [213,214,215,216]. In order to investigate Huntington’s disease and its pathogenesis, different experimental models were designed that express full-length or N-terminal fragments of mutated *HTT*. These models include various transgenic animal models [212,217], models derived from embryonic stem cells [218], and induced pluripotent stem cells [219] of humans with Huntington’s disease. Animal models of Huntington’s disease are mostly transgenic models; however, pharmacological models are also available [220].

Similar to other neurodegenerative disorders, Huntington’s disease is also associated with affected ECS. Expression of CB1R has been downregulated in a subset of neurons of the lateral striatum, cortex and hippocampus in a transgenic animal model of Huntington’s disease [221]. Similar results were observed in the basal ganglia, cerebrum, cerebellum, and brain stem of patients diagnosed with Huntington’s disease [222,223]. Besides the activity and expression of CB1R, CB2R activity also contributes to the disease pathology. CB2R expression of has been elevated in brain tissue from both transgenic animal models of Huntington’s disease and an animal model based on an intra-striatal injection of malonate, the mitochondrial complex II inhibitor [224,225,226].

In mouse models of Huntington’s disease, Bouchard and colleagues demonstrated a beneficial effect of CB2R agonist GW405833 on the life span, motor deficits, synapse loss, and neuroinflammation, while CB2R antagonist SR2 was able to block these effects [224]. CB2R expression has been increased in Huntington’s disease transgenic mouse model and patients with Huntington’s disease, while CB2R deficiency in R6/2 mice led to worsening of disease symptomatology, more pronounced microglia activation and reduction in lifespan [225]. In mice exposed to excitotoxicity, the treatment with CB2R selective agonists was able to alleviate brain edema, loss of striatal neurons and motor symptoms and to reduce neuroinflammation [225]. Moreover, in the malonate model, CB2R activation with agonists protected striatal projection neurons from cell death and decreased TNF-α levels, which were higher due to malonate-induced striatal neuronal death [226]. Contrary to the findings obtained in rodents, human post-mortem study was not able to detect CB2R on either astrocytes or microglia; however, the results suggested their localization on brain vasculature [227]. In summary, regardless of some discrepancies, results emphasize the importance of CB2R in the pathology of Huntington’s disease and as potential therapeutic targets in mitigating or counteracting neurodegeneration.

## 11. CB2R in Amyotrophic Lateral Sclerosis

Amyotrophic lateral sclerosis (ALS) is a progressive neurodegenerative disease of upper and lower motor neurons. It develops rapidly, usually ending in full paralysis and death due to respiratory failure within 2–5 years from diagnosis. ALS is predominantly sporadic. In familial forms, mutations are usually found in *SOD1*, TAR-DNA binding protein-43 (*TDP-43*), and fused in sarcoma (*FUS*) genes, among others [228,229]. Oxidative stress and glutamatergic excitotoxicity, together with the impairment of mitochondrial functions, are recognized as important underlying mechanisms of the disease’s onset and progression [230]. Considering that cannabinoids display direct and indirect antioxidative properties and regulate glutamate release and activity of NMDA receptors [55,231,232,233,234], and taking into account their lipophilic nature and the ability to easily cross the BBB, it comes with no surprise that they have been studied as a potential therapeutic option against ALS.

Altered expression of endocannabinoids and CBR has been found in ALS patients and animal models of the disease. Regarding CB2R, in the spinal cord obtained post-mortem from patients with confirmed ALS, intensive microglial CB2R staining was detected in dorsolateral white matter in the area of the corticospinal tract degeneration [235]. In post-mortem samples of the motor cortex, expression of CB2R was also increased prior to neuronal loss and was associated with reactive activation of astrocytes but not microglia [236]. Similarly, in the spinal cord of the G93A-SOD1 transgenic mice that overexpress mutated human G93A-SOD1 protein and recapitulate many pathological hallmarks of ALS, CB2R mRNA, CB2R binding and CB2R levels were upregulated in a temporal pattern closely following disease progression, whereas changes in CB1R expression were not found [237,238]. Likewise, in the canine form of ALS, which is termed degenerative myelopathy and is caused by mutations in *SOD1*, the CB2R were highly upregulated in the spinal cord, predominantly in activated astrocytes [239].

In TDP-43 transgenic mice (A315T), which is an alternative model of ALS, similarly accompanied by the loss of motor neurons and microglial activation, elevated levels of CB2R mRNA were detected in reactive microglial cells in the spinal ventral horn [240]. Levels of endogenous cannabinoids AEA and 2-AG, as well as levels of AEA synthesizing enzyme NAPE-PLD, were also upregulated in symptomatic mice, probably as a response to neuronal death and microglial activation [238,241,242]. However, elevated levels of endocannabinoids were observed before the appearance of severe motor deficits, suggesting that their sustained enhancement has a protective function in inflamed neural tissue, probably by preventing the toxic effects of microglia on neuronal cells [241]. Specifically, in the presence of an inflammatory stimulus, prolonged stimulation of microglial CB2R suppresses microglial activation and its ability to produce pro-inflammatory mediators, ROS and other neurotoxic markers that ultimately prevent further neuronal damage and reduce neuroinflammation [11,56,57].

Hence, pharmacological interventions targeting the production or metabolism of endocannabinoids, and activation of CBR, CB2R in particular, are appreciated as a reliable therapeutic approach for ALS. Indeed, in animal models of ALS, cannabinoids were effective in ameliorating the progression of the clinical signs of the disease. Data from experimental ALS using nonselective CBR agonists (such as WIN 55,212-2) and selective CB2R agonists suggest the prominent contribution of CB2R and CB2R-mediated suppression of reactive microgliosis in beneficial effects of cannabinoids in ALS. Thus, the daily administration of selective CB2R agonist AM-1241 or WIN 55,212, which began at the time of the symptoms’ appearance, highly extended the survival period of G93A-SOD1 mice [237]. In the same animal model, WIN55,212-2 delayed the progression of the disease when administered after the symptom onset but had no effect on the life span [242]. In G93A-SOD1 mice, AM-1241 was also effective in slowing disease progression when administered after the onset of motoric dysfunctions [243].

Similarly, CBN, a non-psychoactive cannabinoid from marijuana that acts as CB1R and CB2R agonist, delayed disease onset without affecting survival when applied for 12 weeks in G93A-SOD1 mice [244]. In TDP-43 transgenic mice, WIN 55,212-2 showed only trends towards improved motor performance in the rotarod test, recovery of spinal motor neurons, and reduced astrocytic and microglial reactivity, and these effects were partially mediated by CB2R. HU-308, a selective CB2R agonist, exerted more prominent effects. It significantly improved motor deficits and completely preserved motor neurons in the ventral horn, demonstrating the potential of targeting CB2R in ALS [245]. In addition, HU-308 reduced astrocytic activation in the dorsal and ventral horns, and microglial activation in the ventral horn but was without effect or even exacerbated microglial activation in the white matter, perhaps indicating different roles of CB2R on different subpopulations of microglial cells, at least in this ALS model. CB2R in microglial cells located in spinal grey matter were suggested as the most probable molecular target of HU-308, contributing to observed improvements in TDP-43 transgenic mice [245].

The importance of CB2R in maintaining neuronal integrity and survival was also demonstrated in TDP-43 transgenic mice with genetic deletion of *CB2R*. In these animals, the pathological phenotype was significantly accelerated, probably due to the accelerated death of motor neurons, together with the earlier microglial and astrocytic activation and premature mortality. An increase in the magnitude of neuronal death and glial activation was not observed [246]. On the contrary, the genetic deletion of *CB1R* did not modify the disease onset, although it prolonged the life span in G93A-SOD1 mice [242].

However, THC- and CBD-enriched botanical extracts that potentially may cover many pharmacological targets, including the CB2R activation, produced only small improvements in G93A-SOD1 mice, particularly in females, when applied at the appearance of the first motor symptoms. Its administration slightly delayed the progression of neurological deficits and tended to increase the survival period, despite the highly elevated CB2R expression [238]. Similarly, moderate effects have been observed following cannabis use in ALS patients. They reported short-term relief for depression, pain, loss of appetite, spasticity and drooling, but there was no improvement in speech and swallowing dysfunctions [247]. Hence, despite many promising findings in animal models, further studies, particularly clinical studies, are needed before supporting the therapeutic benefits of cannabinoids and CB2R targeting in ALS patients [246,248].

## 12. CB2R in Multiple Sclerosis

Multiple sclerosis is a chronic autoimmune inflammatory disease of the CNS. It is characterized by the BBB disruption, infiltration of the peripheral immune cells into the brain, microglial activation and neuroinflammation, axonal demyelination, neurodegeneration, and consequently, the appearance of neurological dysfunctions. The precise mechanism driving the immune impairment is not fully understood. It is considered that interactions between distinct genetic and environmental factors contribute to the autoimmune response mediated predominantly by myelin-specific Th17 and Th1 cells [249,250]. Experimental autoimmune encephalomyelitis (EAE) is an animal model widely used for studying immunological and neuropathological mechanisms of the disease onset and progression, as well as the efficacy of various pharmacological agents in combating multiple sclerosis. In mice, EAE is induced by exposure to myelin antigens, usually myelin oligodendrocytes glycoprotein (MOG) and proteolipid protein peptide (PLP) [251,252].

It has been shown that CB1R and CB2R play a protective role in EAE. In general, the neuronal CB1R signalling controls the neurodegenerative damage and is more relevant in the context of neuroprotection, whereas CB2R activation modulates immune response and is related to the anti-inflammatory effects of cannabinoids [253,254,255]. By targeting CB2R, cannabinoids suppress the production of pro-inflammatory cytokines, as well as ROS and NOS in microglial cells, and prevent proliferation, migration, recruitment and antigen-presenting properties of immune cells [251,256]. Accordingly, CB1R and CB2R deficiency exacerbates EAE pathogenesis. In particular, CB2R knock-out mice have deteriorated clinical scores, accompanied by more severe axonal loss, infiltration of the CD4+ lymphocytes, microglial activation and neuroinflammation, and show reduced susceptibility to beneficial effects of cannabinoids [251,253,255]. Vice versa, selective CB2R activation reduces EAE symptoms, axonal loss and microglial activation [251,252].

As in other neuroinflammatory and neurodegenerative diseases, CB1R and CB2R expression is altered in both multiple sclerosis and EAE. One study has shown that patients with multiple sclerosis have increased CB2R expression in B cells but not in T cells or natural killer cells, together with increased AEA levels in all three cell populations [257]. Similarly, in the post-mortem human spinal cord, CB2R levels were increased in affected regions, together with the increased number of CB2-immunoreactive microglial cells [235]. EAE similarly induces a sustained increase of the ECS components in activated microglial cells, particularly of CB2R, although levels of CB1R, FAAH and MAGL were also higher in the spinal cord microglial cells [251,258]. Overall, these studies indicate that microglial activation in multiple sclerosis is accompanied by a prominent increase of CB2R, implying their important role in the inflammatory response.

Furthermore, an association has been observed between multiple sclerosis and the single-nucleotide polymorphism Q63R in the *CNR2* gene coding for the CB2R functional variation [259]. This polymorphism compromises CB2R activation and reduces WIN 55,212-2 and 2-AG signalling, suggesting that it may affect response to drug treatment [260]. As 2-AG is likely the major endogenous ligand of CB2R, this suggests that the immunosuppressive effects of 2-AG will be less efficient in Q63R carriers, probably contributing to their higher risk of developing multiple sclerosis [59,260].

Many reports demonstrated the beneficial effects of cannabinoids in multiple sclerosis based on their immunosuppressive, neuroprotective, remyelinating and analgesic effects [261,262,263]. For example, in Theiler’s virus infection of the CNS, which induces an immune-mediated demyelinating disease resembling multiple sclerosis, synthetic CBR agonists WIN 55,212–2 (nonselective CB1/CB2 agonist), ACEA (selectivity over CB1R), and JWH-015 (selectivity over CB2R) improved motor functions, reduced microglial activation and number of CD4+ infiltrating T-cells in the spinal cord, and promoted remyelination in infected animals, clearly suggesting the therapeutic potential of targeting ECS in multiple sclerosis [264]. Likewise, THC, which is a CB1R and CB2R agonist, inhibited the severity of EAE and delayed disease onset [253]. However, as the beneficial effects of nonselective cannabinoids are usually accompanied by the undesirable psychoactive effects mediated by CB1R, the major pharmacological approaches against multiple sclerosis are directed towards selective CB2R agonists that target the CB2R on microglial cells and consequently inhibit the release of various pro-inflammatory mediators and cytokine-mediated demyelination process [265]. Thus, animals treated with a Gp1a (N-(piperidin-1-yl)-1-(2,4-dichlorophenyl)-1,4-dihydro-6-methylindeno[1,2-c]pyrazole-3-carboxamide), the highly selective CB2R agonist, had a lower incidence of EAE, delayed EAE onset, and reduced clinical score, suggesting an effect of Gp1a on the attenuation of the EAE development.

Moreover, Gp1a reduced the number of CD4+ T-cells, Th1 and Th17 cells, i.e., suppressed differentiation of Th1 and Th17 cells in peripheral immune tissue, decreased infiltration of immune cells, and reduced microglial activation. Moreover, applied at a later time point, when the CD4+ T-cells were already activated and differentiated, Gp1a promoted recovery by decreasing the expression of pro-inflammatory cytokines, chemokines, adhesion molecules and iNOS. Delayed Gp1a treatment also reduced the infiltration of immune cells and prevented the accumulation of pathogenic T-cells inside the CNS, and reduced the demyelination and axonal damage that contributed to better clinical recovery [252]. Likewise, daily injection of HU-308, a selective CB2R agonist, even if started on the day of the maximal disease score, improved the EAE symptoms. HU-308 reduced the proliferation of resident microglial cells and by reducing the expression of chemoattractant ligands and their receptors, it inhibited the recruitment of the myeloid progenitor cells that can replenish microglial cells [251].

Yet another class of compounds, 1,8-naphthyridine, and its pyridine and quinoline derivatives, that display higher CB2R selectivity in comparison to CB1R exerted beneficial effects. In the activated lymphocytes of patients with multiple sclerosis, these compounds demonstrated immuno-modulatory and anti-inflammatory effects that were partially mediated by CB2R. They suppressed cell proliferation and down-regulated myelin basic protein (MBP)-induced activation of Akt and NF-κB, production of TNF-α, and expression of activation markers such as COX-2, altogether supporting the potential of these compounds in the therapy of multiple sclerosis [266]. Similarly, (−)-β-caryophyllene (BCP), a dietary CB2 selective agonist, demonstrated beneficial effects against MOG-induced murine EAE. In MOG-primed T-cells, BCP upregulated production of IL-10 and reduced levels of IFN-γ, whereas in animals, it prevented motor paralysis and weight loss, inhibited microglial activation, oxidative injury and axonal demyelination, and decreased the number and activation of CD4+ and CD8+ T-cells in peripheral lymphoid tissue, likely indicating that BCP stimulates infiltration and differentiation of Treg and inhibits myelin-specific Th1 cells acting at CB2R [267].

Regarding endocannabinoids, it has been shown that AEA inhibits proliferation and cytokine release from human T lymphocytes, including IL-17 production from the CD4+ Th17 cells, mainly acting via CB2R [256]. In agreement with these findings, mice lacking FAAH, which consequently have highly increased AEA levels, developed less severe symptoms of EAE [255]. Finally, botanical extracts enriched with THC and CBD in a 1:1 molecular ratio, applied as oromucosal spray (Nabiximols, Sativex), have been approved for alleviating pain and spasticity in patients with multiple sclerosis [268,269]. However, despite the recognized contribution of CB2R signalling in immunosuppressive and beneficial effects of cannabinoids, phytocannabinoids in particular, pharmacological and genetic approaches in various animal models of multiple sclerosis brought to light that not all anti-inflammatory effects of cannabinoids are mediated by CB1R and CB2R. For example, CBD, a weak agonist of CB1R and CB2R, ameliorated clinical signs of the MOG-induced EAE in C57BL/6 mice when administered during the disease onset. It reduced axonal damage, microglial activation and proliferation and infiltration of T cells in the spinal cord of EAE mice, but the effect was not mediated via CB2R [261]. Hence, although selective CB2R agonists represent an attractive pharmacological intervention for immune regulation in multiple sclerosis, particularly if considering that they are devoid of psychoactive effects, further clinical validation is needed to provide better insight into the potential of targeting CB2R in multiple sclerosis.

## 13. Conclusions and Future Perspectives

The ECS has been extensively studied and recognized for its therapeutic role in recent decades, due to its involvement in numerous physiological and pathological processes [103]. Significant progress in cannabinoid research, as well as the detection of CB2R expression in the brain, have directed investigation focus toward CR2R, as potential targets for treatment of neurodegenerative proteinopathies. Specifically, in contrast to CB1R, which were found in most brain areas, initial studies detected CB2R expression mostly in peripheral organs and tissues with immune function, where they have been involved in cell proliferation and migration, cytokine production and phagocytic activity, apoptosis and immunosuppression. However, later studies identified the presence of CB2R in the brain, in reactive microglia, activated astrocytes, oligodendrocytes, and in some neuronal subpopulations [270].

In addition, CB2R are found to be highly upregulated in the brain samples of patients suffering from neurodegenerative disorders, such as Alzheimer’s disease, Parkinson’s disease or Huntington’s disease, amyotrophic lateral sclerosis and multiple sclerosis, as well as in the corresponding animal models [271]. Since their expression is selectively increased in activated microglia that are recruited to the sites of neurodegeneration, CB2R represent promising preventive/therapeutic candidates in neurodegenerative proteinopathies, which involve a neuroinflammatory component. Preserving healthy neurons or rescuing damaged neurons may be achieved by selecting the right agonist or allosteric modulator of CB2R. Although various selective CB2R compounds have been investigated, only some of them have reached clinical trials. In many preclinical studies, selective CBR2 agonists have been shown to exert immunomodulatory and neuroprotective actions, to attenuate neuroinflammation by suppressing microglial reactivity and expression of pro-inflammatory cytokines, decrease the production of misfolded protein aggregates and facilitate their clearance, reduce excitotoxicity and oxidative/nitrosative stress, as well as to improve cognitive functions [8,9].

However, clinical data are needed to reveal the full potential of cannabinoids against various proteinopathies, particularly for the most common Alzheimer’s disease and Parkinson’s disease, for which there is still no satisfactory and disease-modifying therapy. Besides THC and CBD, there are other phytocannabinoids whose neuroprotective potential has not yet been assessed, neither via CB2R nor through other receptor targets.

Even though CB2R agonists offer promising therapeutic potential, their translation into clinical application depends on overcoming some limitations. Since it is known that molecular changes underlying neurodegenerative proteinopathies usually begin years before symptoms manifestation, in order to achieve potential neuroprotective and immunomodulatory effects, drugs specifically targeting CB2R should probably be administered for a prolonged time before the exacerbation of these diseases. Therefore, studies on the long-term effects of CB2R-targeted drug therapy, especially in the early phases of disease development are needed. Besides the treatment time, identifying an effective and safe drug dosage is also challenging, especially since the information regarding the efficacy or toxicity of such compounds in humans is lacking. It is possible that the non-specific binding of CB2R ligands to other receptors (TRPV1, PPARs) in the CNS can reduce the drug concentrations targeting CB2R. On the other hand, since CB2R are abundant in the peripheral immune system, CB2R agonists may exhibit some chronic side effects, such as immune suppression [272]. Moreover, considering that patients with neurodegenerative disorders take multiple medications and CB2R agonists could be included as an additional therapeutic option, further investigations evaluating drug-drug interactions are required.

Nevertheless, since CB2R recognize fewer endogenous ligands compared to CBR1, and have restricted neuronal expression, drugs targeting CBR2 are considered safer and well tolerated in clinical applications, without unwanted psychoactive side effects like anxiety, mood disturbances, psychosis, cognitive impairment, memory and attention deficits, which are observed after CB1R activation [273]. However, to avoid the potential peripheral side effects of CB2R agonists, it would be desirable to use brain-targeted delivery systems. Another advantage is that CB2R have remarkable functional selectivity and can specifically activate different intracellular signaling pathways with different ligands or using different ligand doses, further expanding the possibility of selectively targeting CB2R [274]. The ligand-biased signaling profiles of CB2R ligands continue to be investigated and, upon validation, they could open new therapeutic directions. In the future, the discovery of positive and negative CB2R allosteric modulators may both finetune the CB2R response and minimize the side effects.

However, the search to find novel CB2R therapeutics for neurodegenerative proteinopathies has been challenging. One of the challenges in CB2R-based drug design is the complex CB2R pharmacological characterization due to the lipophilic nature of many cannabinoids that have to reach the receptor-binding site located deeply within the transmembrane domain through the lipid bilayer of the plasma membrane [103]. Since CBR may interact and give rise to CB1R-CB2R heteromers [275], another therapeutic strategy might be to target CB2R homo- or heterodimers with homobivalent and heterobivalent ligands. However, the design and synthesis of bivalent drugs for CB2R is very complex since they showed to be weak antagonists/inverse agonists of CB1R and CB2R, in contrast to the monomeric parent molecule, which was a selective agonist for CB2R [118]. The main advantage of using drugs that preferentially act on cells expressing heteromers would be the reduction of side effects. Another potential therapeutic strategy involves compounds, which exert dual actions on the ECS, such as 4′-O-methylhokiol, which acts as both a CB2R modulator and COX-2 substrate-specific inhibitor [116].

Further studies of the CB2R and their downstream signaling pathway are needed to assess the full neuroprotective potential of selective drugs able to modulate the CB2R, which are up-regulated in activated astrocytes and reactive microglia in response to neuroinflammatory processes that occur in neurodegenerative disorders. Hopefully, various currently available technologies and tools, including the -omics methods, bioinformatics and systems biology approaches, will help to utilize the complexity of ECS and CB2R in order to discover novel potential therapeutic opportunities for neurodegenerative proteinopathies. For instance, next-generation sequencing (NGS) has been shown as a powerful diagnostic tool that can collect information about neurodegenerative diseases at genomic, transcriptomic and epigenetic levels [276]. As in the case of other CNS pathologies with an important oxidative stress and inflammation component [277,278], this method could contribute to a better understanding of their contribution in the pathogenesis of neurodegenerative proteinopathies, as potential targets of selective CB2R- based therapeutics.

## Figures and Tables

**Table 1:** Misfolded and aggregated proteins in neurodegenerative proteinopathies.

Neurodegenerative Proteinopathy	Misfolded and Aggregated Protein(S)
Alzheimer’s disease (AD)	Amyloid beta (Aβ) peptide, tau
Parkinson’s disease (PD)	α-synuclein
Huntington´s disease (HD)	Mutant huntingtin (mHtt)
Multiple sclerosis (MS)	Bassoon presynaptic cytomatrix protein
Amyotrophic lateral sclerosis (ALS)	Mutant superoxide dismutase 1 (mSOD1), TAR DNA-binding protein 43 (TDP-43) and fused-in-sarcoma (FUS) protein
Dementia with Lewy bodies (DLB)	α-synuclein
Frontotemporal lobar degeneration (FTLD)	FTLD-tau, FTLD-TDP, FTLD-FUS
Creutzfeldt–Jakob disease (CJD)	Protease-resistant cellular prion protein (PrP^Sc^)

## Data Availability

Not applicable.

## References

[1] Noor A., Zafar S., Zerr I. (2021). Neurodegenerative Proteinopathies in the Proteoform Spectrum—Tools and Challenges. Int. J. Mol. Sci..

[2] Knowles T.P.J., Vendruscolo M., Dobson C.M. (2014). The amyloid state and its association with protein misfolding diseases. Nat. Rev. Mol. Cell Biol..

[3] Ciccocioppo F., Bologna G., Ercolino E., Pierdomenico L., Simeone P., Lanuti P., Pieragostino D., Del Boccio P., Marchisio M., Miscia S. (2020). Neurodegenerative diseases as proteinopathies-driven immune disorders. Neural Regen. Res..

[4] Winklhofer K.F., Tatzelt J., Haass C. (2008). The two faces of protein misfolding: Gain- and loss-of-function in neurodegenerative diseases. EMBO J..

[5] Sami N., Rahman S., Kumar V., Zaidi S., Islam A., Ali S., Ahmad F., Hassan M.I. (2017). Protein aggregation, misfolding and consequential human neurodegenerative diseases. Int. J. Neurosci..

[6] Soto C., Pritzkow S. (2018). Protein misfolding, aggregation, and conformational strains in neurodegenerative diseases. Nat. Neurosci..

[7] Chitnis T., Weiner H.L. (2017). CNS inflammation and neurodegeneration. J. Clin. Investig..

[8] Pagano C., Navarra G., Coppola L., Avilia G., Bifulco M., Laezza C. (2022). Cannabinoids: Therapeutic Use in Clinical Practice. Int. J. Mol. Sci..

[9] Kibret B.G., Ishiguro H., Horiuchi Y., Onaivi E.S. (2022). New Insights and Potential Therapeutic Targeting of CB2 Cannabinoid Receptors in CNS Disorders. Int. J. Mol. Sci..

[10] García-Gutiérrez M.S., Navarrete F., Gasparyan A., Manzanares J. (2021). Therapeutic potential of the cannabinoid receptor 2 in neuropsychiatry. Explor. Neuroprot. Ther..

[11] Komorowska-Müller J.A., Schmöle A.C. (2021). CB2 Receptor in Microglia: The Guardian of Self-Control. Int. J. Mol. Sci..

[12] Schattling B., Engler J.B., Volkmann C., Rothammer N., Woo M.S., Petersen M., Winkler I., Kaufmann M., Rosenkranz S.C., Fejtova A. (2019). Bassoon proteinopathy drives neurodegeneration in multiple sclerosis. Nat. Neurosci..

[13] Sweeney P., Park H., Baumann M., Dunlop J., Frydman J., Kopito R., McCampbell A., Leblanc G., Venkateswaran A., Nurmi A. (2017). Protein misfolding in neurodegenerative diseases: Implications and strategies. Transl. Neurodegener..

[14] Hartl U.F. (2017). Protein misfolding diseases. Annu. Rev. Biochem..

[15] Marsh A.P. (2019). Molecular mechanisms of proteinopathies across neurodegenerative disease: A review. Neurol. Res. Pract..

[16] Ciechanover A., Kwon Y.T. (2017). Protein quality control by molecular chaperones in neurodegeneration. Front. Neurosci..

[17] Hayashida N., Fujimoto M., Tan K., Prakasam R., Shinkawa T., Li L., Ichikawa H., Takii R., Nakai A. (2010). Heat shock factor 1 ameliorates proteotoxicity in cooperation with the transcription factor NFAT. EMBO J..

[18] Hipp M.S., Park S.H., Hartl U.U. (2014). Proteostasis impairment in protein-misfolding and aggregation diseases. Trends Cell Biol..

[19] Nonaka T., Hasegawa M. (2009). A cellular model to monitor proteasome dysfunction by α-synuclein. Biochemistry.

[20] Finley D. (2009). Recognition and processing of ubiquitin-protein conjugates by the proteasome. Annu. Rev. Biochem..

[21] Brundin P., Melki R., Kopito R. (2010). Prion-like transmission of protein aggregates in neurodegenerative diseases. Nat. Rev. Mol. Cell Biol..

[22] Brettschneider J., Del Tredici K., Lee V.M.Y., Trojanowski J.Q. (2015). Spreading of pathology in neurodegenerative diseases: A focus on human studies. Nat. Rev. Neurosci..

[23] Galpern W.R., Lang A.E. (2006). Interface between tauopathies and synucleinopathies: A tale of two proteins. Ann. Neurol..

[24] Gidalevitz T., Krupinski T., Garcia S., Morimoto R.I. (2009). Destabilizing protein polymorphisms in the genetic background direct phenotypic expression of mutant SOD1 toxicity. PLoS Genet..

[25] Wu J.W., Hussaini S.A., Bastille I.M., Rodriguez G.A., Mrejeru A., Rilett K., Sanders D.W., Cook C., Fu H., Boonen R.A. (2016). Neuronal activity enhances tau propagation and tau pathology in vivo. Nat. Neurosci..

[26] Lee J.G., Takahama S., Zhang G., Tomarev S.I., Ye Y. (2016). Unconventional secretion of misfolded proteins promotes adaptation to proteasome dysfunction in mammalian cells. Nat. Cell Biol..

[27] Shankar G.M., Li S., Mehta T.H., Garcia-Munoz A., Shepardson N.E., Smith I., Brett F.M., Farrell M.A., Rowan M.J., Lemere C.A. (2008). Amyloid-β protein dimers isolated directly from Alzheimer’s brains impair synaptic plasticity and memory. Nat. Med..

[28] Kopeikina K.J., Hyman B.J., Spires-Jones T.L. (2012). Soluble forms of tau are toxic in Alzheimer’s disease. Transl. Neurosci..

[29] Ingelsson M. (2016). Alpha-synuclein oligomers-neurotoxic molecules in Parkinson’s disease and other Lewy body disorders. Front. Neurosci..

[30] Majd S., Power J.H., Grantham H.J.M. (2015). Neuronal response in Alzheimer’s and Parkinson’s disease: The effect of toxic proteins on intracellular pathway. BMC Neurosci..

[31] Ben Haim L., Carrillo-de Sauvage M.A., Ceyzériat K., Escartin C. (2015). Elusive roles for reactive astrocytes in neurodegenerative diseases. Front. Cell. Neurosci..

[32] Jiang T., Sun Q., Chen S. (2016). Oxidative stress: A major pathogenesis and potential therapeutic target of antioxidative agents in Parkinson’s disease and Alzheimer’s disease. Prog. Neurobiol..

[33] Angelova P.R., Abramov A.Y. (2017). Alpha-synuclein and beta-amyloid—Different targets, same players: Calcium, free radicals and mitochondria in the mechanism of neurodegeneration. Biochem. Biophys. Res. Commun..

[34] Taylor R.C., Dillin A. (2011). Aging as an event of proteostasis collapse. Cold Spring Harb. Perspect. Biol..

[35] Höhn A., Weber D., Jung T., Ott C., Hugo M., Kochlik B., Kehm R., König J., Grune T., Castro J.P. (2017). Happily (n)ever after: Aging in the context of oxidative stress, proteostasis loss and cellular senescence. Redox Biol..

[36] Heppner F.L., Ransohoff R.M., Becher B. (2015). Immune attack: The role of inflammation in Alzheimer disease. Nat. Rev. Neurosci..

[37] Chakrabarty P., Li A., Ceballos-Diaz C., Eddy J.A., Funk C.C., Moore B., DiNunno N., Rosario A.M., Cruz P.E., Verbeecket C. (2015). IL-10 Alters Immunoproteostasis in APP Mice, Increasing Plaque Burden and Worsening Cognitive Behavior. Neuron.

[38] Guillot-Sestier M.V., Doty K.R., Gate D., Rodriguez J., Leung B.P., Rezai-Zadeh K., Town T. (2015). Il10 deficiency rebalances innate immunity to mitigate Alzheimer-like pathology. Neuron.

[39] Deleidi M., Jäggle M., Rubino G. (2015). Immune ageing, dysmetabolism and inflammation in neurological diseases. Front. Neurosci..

[40] Currais A., Fischer W., Maher P., Schubert D. (2017). Intraneuronal protein aggregation as a trigger for inflammation and neurodegeneration in the aging brain. FASEB J..

[41] Battista N., Di Tommaso M., Bari M., Maccarrone M. (2012). The endocannabinoid system: An overview. Front. Behav. Neurosci..

[42] Aizpurua-Olaizola O., Elezgarai I., Rico-Barrio I., Zarandona I., Etxebarria N., Usobiaga A. (2017). Targeting the endocannabinoid system: Future therapeutic strategies. Drug Discov. Today.

[43] Lu H.C., Mackie K. (2021). Review of the Endocannabinoid System. Biol Psychiatry. Cogn. Neurosci. Neuroimaging.

[44] Chanda D., Neumann D., Glatz J.F.C. (2019). The endocannabinoid system: Overview of an emerging multi-faceted therapeutic target. Prostaglandins Leukot. Essent. Fat. Acids.

[45] Lu H.C., MacKie K. (2016). An introduction to the endogenous cannabinoid system. Biol. Psychiatry.

[46] Glass M., Dragunow M., Faull R.L.M. (1997). Cannabinoid receptors in the human brain: A detailed anatomical and quantitative autoradiographic study in the fetal, neonatal and adult human brain. Neuroscience.

[47] Biegon A., Kerman I.A. (2001). Autoradiographic study of pre- and postnatal distribution of cannabinoid receptors in human brain. Neuroimage.

[48] Morris G., Walder K., Kloiber S., Amminger P., Berk M., Bortolasci C.C., Maes M., Puri B.K., Carvalho A.F. (2021). The endocannabinoidome in neuropsychiatry: Opportunities and potential risks. Pharmacol. Res..

[49] Di Iorio G., Lupi M., Sarchione F., Matarazzo I., Santacroce R., Petruccelli F., Martinotti G., Di Giannantonio M. (2013). The Endocannabinoid System: A Putative Role in Neurodegenerative Diseases. Int. J. High Risk Behav. Addict..

[50] Marco E.M., García-Gutiérrez M.S., Bermúdez-Silva F.J., Moreira F.A., Guimarães F., Manzanares J., Viveros M.P. (2011). Endocannabinoid system and psychiatry: In search of a neurobiological basis for detrimental and potential therapeutic effects. Front. Behav. Neurosci..

[51] Galiègue S., Mary S., Marchand J., Dussossoy D., Carrière D., Carayon P., Bouaboula M., Shire D., Le Fur G., Casellas P. (1995). Expression of Central and Peripheral Cannabinoid Receptors in Human Immune Tissues and Leukocyte Subpopulations. Eur. J. Biochem..

[52] Zou S., Kumar U. (2018). Cannabinoid Receptors and the Endocannabinoid System: Signaling and Function in the Central Nervous System. Int. J. Mol. Sci..

[53] Howlett A.C. (2002). The cannabinoid receptors. Prostaglandins Other Lipid Mediat..

[54] Paloczi J., Varga Z.V., Hasko G., Pacher P. (2017). Neuroprotection in Oxidative Stress-Related Neurodegenerative Diseases: Role of Endocannabinoid System Modulation. Antioxid. Redox Signal..

[55] Tadijan A., Vlasic I., Vlainic J., Dikic D., Orsolic N., Jazvinscak Jembrek M. (2022). Intracellular Molecular Targets and Signaling Pathways Involved in Antioxidative and Neuroprotective Effects of Cannabinoids in Neurodegenerative Conditions. Antioxidants.

[56] Ehrhart J., Obregon D., Mori T., Hou H., Sun N., Bai Y., Klein T., Fernandez F., Tan J., Shytle R.D. (2005). Stimulation of cannabinoid receptor 2 (CB2) suppresses microglial activation. J. Neuroinflamm..

[57] Ma L., Jia J., Liu X., Bai F., Wang Q., Xiong L. (2015). Activation of murine microglial N9 cells is attenuated through cannabinoid receptor CB2 signaling. Biochem. Biophys. Res. Commun..

[58] Cristino L., Bisogno T., Di Marzo V. (2020). Cannabinoids and the expanded endocannabinoid system in neurological disorders. Nat. Rev. Neurol..

[59] Pertwee R.G., Howlett A.C., Abood M.E., Alexander S.P.H., Di Marzo V., Elphick M.R., Greasley P.J., Hansen H.S., Kunos G., Mackie K. (2010). International Union of Basic and Clinical Pharmacology. LXXIX. Cannabinoid receptors and their ligands: Beyond CB1 and CB2. Pharmacol. Rev..

[60] Joshi N., Onaivi E.S. (2019). Endocannabinoid System Components: Overview and Tissue Distribution. Recent Advances in Cannabinoid Physiology and Pathology.

[61] Bisogno T., Berrendero F., Ambrosino G., Cebeira M., Ramos J.A., Fernandez-Ruiz J.J., Di Marzo V. (1999). Brain regional distribution of endocannabinoids: Implications for their biosynthesis and biological function. Biochem. Biophys. Res. Commun..

[62] Felder C.C., Nielsen A., Briley E.M., Palkovits M., Priller J., Axelrod J., Nguyen D.N., Richardson J.M., Riggin R.M., Koppel G.A. (1996). Isolation and measurement of the endogenous cannabinoid receptor agonist, anandamide, in brain and peripheral tissues of human and rat. FEBS Lett..

[63] Castillo P.E., Younts T.J., Chávez A.E., Hashimotodani Y. (2012). Endocannabinoid Signaling and Synaptic Function. Neuron.

[64] Katona I., Freund T.F. (2008). Endocannabinoid signaling as a synaptic circuit breaker in neurological disease. Nat. Med..

[65] Pacher P., Batkai S., Kunos G. (2006). The Endocannabinoid System as an Emerging Target of Pharmacotherapy. Pharmacol. Rev..

[66] Murataeva N., Straiker A., MacKie K. (2014). Parsing the players: 2-arachidonoylglycerol synthesis and degradation in the CNS. Br. J. Pharmacol..

[67] Jung K.M., Sepers M., Henstridge C.M., Lassalle O., Neuhofer D., Martin H., Ginger M., Frick A., DiPatrizio N.V., Mackie K. (2012). Uncoupling of the endocannabinoid signalling complex in a mouse model of fragile X syndrome. Nat. Commun..

[68] Kano M., Ohno-Shosaku T., Hashimotodani Y., Uchigashima M., Watanabe M. (2009). Endocannabinoid-mediated control of synaptic transmission. Physiol. Rev..

[69] Glaser S.T., Abumrad N.A., Fatade F., Kaczocha M., Studholme K.M., Deutsch D.G. (2003). Evidence against the presence of an anandamide transporter. Proc. Natl. Acad. Sci. USA.

[70] McFarland M.J., Porter A.C., Rakhshan F.R., Rawat D.S., Gibbs R.A., Barker E.L. (2004). A role for caveolae/lipid rafts in the uptake and recycling of the endogenous cannabinoid anandamide. J. Biol. Chem..

[71] Huang H., McIntosh A.L., Martin G.G., Landrock D., Chung S., Landrock K.K., Dangott L.J., Li S., Kier A.B., Schroeder F. (2016). FABP1: A Novel Hepatic Endocannabinoid and Cannabinoid Binding Protein. Biochemistry.

[72] Blankman J.L., Simon G.M., Cravatt B.F. (2007). A Comprehensive Profile of Brain Enzymes that Hydrolyze the Endocannabinoid 2-Arachidonoylglycerol. Chem. Biol..

[73] Berry A.J., Zubko O., Reeves S.J., Howard R.J. (2020). Endocannabinoid system alterations in Alzheimer’s disease: A systematic review of human studies. Brain Res..

[74] Behl T., Kaur G., Bungau S., Jhanji R., Kumar A., Mehta V., Zengin G., Brata R., Hassan S.S.U., Fratila O. (2020). Distinctive evidence involved in the role of endocannabinoid signalling in parkinson’s disease: A perspective on associated therapeutic interventions. Int. J. Mol. Sci..

[75] Reddy V., Grogan D., Ahluwalia M., Salles É.L., Ahluwalia P., Khodadadi H., Alverson K., Nguyen A., Raju S.P., Gaur P. (2020). Targeting the endocannabinoid system: A predictive, preventive, and personalized medicine-directed approach to the management of brain pathologies. EPMA J..

[76] Jones É., Vlachou S. (2020). A Critical Review of the Role of the Cannabinoid Compounds Δ9-Tetrahydrocannabinol (Δ9-THC) and Cannabidiol (CBD) and their Combination in Multiple Sclerosis Treatment. Molecules.

[77] Fakhoury M. (2017). Role of the Endocannabinoid System in the Pathophysiology of Schizophrenia. Mol. Neurobiol..

[78] Papagianni E.P., Stevenson C.W. (2019). Cannabinoid Regulation of Fear and Anxiety: An Update. Curr. Psychiatry Rep..

[79] Huang W.J., Chen W.W., Zhang X. (2016). Endocannabinoid system: Role in depression, reward and pain control (Review). Mol. Med. Rep..

[80] Davis J., Moylan S., Harvey B.H., Maes M., Berk M. (2014). Neuroprogression in schizophrenia: Pathways underpinning clinical staging and therapeutic corollaries. Aust. N. Z. J. Psychiatry.

[81] Berk M., Kapczinski F., Andreazza A.C., Dean O.M., Giorlando F., Maes M., Yücel M., Gama C.S., Dodd S., Dean B. (2011). Pathways underlying neuroprogression in bipolar disorder: Focus on inflammation, oxidative stress and neurotrophic factors. Neurosci. Biobehav. Rev..

[82] Di Marzo V. (2008). Targeting the endocannabinoid system: To enhance or reduce?. Nat. Rev. Drug Discov..

[83] Hanuš L.O., Meyer S.M., Muñoz E., Taglialatela-Scafati O., Appendino G. (2016). Phytocannabinoids: A unified critical inventory. Nat. Prod. Rep..

[84] Brown J.D., Rivera Rivera K.J., Hernandez L.Y.C., Doenges M.R., Auchey I., Pham T., Goodin A.J. (2021). Natural and Synthetic Cannabinoids: Pharmacology, Uses, Adverse Drug Events, and Drug Interactions. J. Clin. Pharmacol..

[85] Bie B., Wu J., Foss J.F., Naguib M. (2018). An overview of the cannabinoid type 2 receptor system and its therapeutic potential. Curr. Opin. Anaesthesiol..

[86] Ruhaak L.R., Felth J., Karlsson P.C., Rafter J.J., Verpoorte R., Bohlin L. (2011). Evaluation of the Cyclooxygenase Inhibiting Effects of Six Major Cannabinoids Isolated from Cannabis sativa. Biol. Pharm. Bull..

[87] Pisanti S., Malfitano A.M., Ciaglia E., Lamberti A., Ranieri R., Cuomo G., Abate M., Faggiana G., Proto M.C., Fiore D. (2017). Cannabidiol: State of the art and new challenges for therapeutic applications. Pharmacol. Ther..

[88] Thakur G.A., Duclos R.I., Makriyannis A. (2005). Natural cannabinoids: Templates for drug discovery. Life Sci..

[89] Morales P., Hurst D.P., Reggio P.H. (2017). Molecular Targets of the Phytocannabinoids: A Complex Picture. Prog. Chem. Org. Nat. Prod..

[90] Schlatter J., Atta U.-R. (2014). Synthetic Cannabinoids: Synthesis and Biological Activities. Studies in Natural Products Chemistry.

[91] Abate G., Uberti D., Tambaro S. (2021). Potential and Limits of Cannabinoids in Alzheimer’s Disease Therapy. Biology.

[92] Fernández-Ruiz M., Romero J., Velasco G., Tolon R.M., Ramos J.A., Guzman M. (2007). Cannabinoid CB2 receptor: A new target for controlling neural cell survival?. Trends Pharmacol. Sci..

[93] Munro S., Thomas K.L., Abu-Shaar M. (1993). Molecular characterization of a peripheral receptor for cannabinoids. Nature.

[94] Schatz A.R., Lee M., Condie R.B., Pulaski J.T., Kaminski N.E. (1997). Cannabinoid receptors CB1 and CB2: A characterization of expression and adenylate cyclase modulation within the immune system. Toxicol. Appl. Pharmacol..

[95] Polini B., Cervetto C., Carpi S., Pelassa S., Gado F., Ferrisi R., Bertini S., Nieri P., Marcoli M., Manera C. (2020). Positive Allosteric Modulation of CB1 and CB2 Cannabinoid Receptors Enhances the Neuroprotective Activity of a Dual CB1R / CB2R Orthosteric Agonist. Life.

[96] Brown S.M., Wager-Miller J., Mackie K. (2002). Cloning and Molecular Characterization of the Rat CB2 Cannabinoid Receptor. Biochim. Biophys. Acta.

[97] Chen D.J., Gao M., Gao F.F., Su Q.X., Wu J. (2017). Brain cannabinoid receptor 2: Expression, function and modulation. Acta Pharmacol. Sin..

[98] Onaivi E.S., Ishiguro H., Gu S., Liu Q.R. (2012). CNS effects of CB2 cannabinoid receptors: Beyond neuro-immuno-cannabinoid activity. J. Psychopharmacol..

[99] Buckley N.E., McCoy K.L., Mezey E., Bonner T., Zimmer A., Felder C.C., Glass M., Zimmer A. (2000). Immunomodulation by cannabinoids is absent in mice deficient for the cannabinoid CB2 receptor. Eur. J. Pharmacol..

[100] Gong J.P., Onaivi E.S., Ishiguro H., Liu Q.R., Tagliaferro P.A., Brusco A., Uhl G.R. (2006). Cannabinoid CB2 receptors: Immunohistochemical localization in rat brain. Brain Res..

[101] Onaivi E.S., Ishiguro H., Gong J.P., Patel S., Meozzi P.A., Myers L., Perchuk A., Mora Z., Tagliaferro P.A., Gardner E. (2008). Functional expression of brain neuronal CB2 cannabinoid receptors are involved in the effects of drugs of abuse and in depression. Ann. N. Y. Acad. Sci..

[102] Wang M., Liu H., Ma Z. (2022). Roles of the Cannabinoid System in the Basal Ganglia in Parkinson’s Disease. Front. Cell. Neurosci..

[103] Di Marzo V. (2018). New approaches and challenges to targeting the endocannabinoid system. Nat. Rev. Drug Discov..

[104] Brusco A., Tagliaferro P., Saez T., Onaivi E.S. (2008). Postsynaptic localization of CB2 cannabinoid receptors in the rat hippocampus. Synapse.

[105] Onaivi E.S., Ishiguro H., Gong J.P., Patel S., Perchuk A., Meozzi P.A., Myers L., Mora Z., Tagliaferro P., Gardner E. (2006). Discovery of the presence and functional expression of cannabinoid CB2 receptors in brain. Ann. N. Y. Acad. Sci..

[106] Chin C.L., Tovcimak A.E., Hradil V.P., Seifert T.R., Hollingsworth P.R., Chandran P., Zhu C.Z., Gauvin D., Pai M., Wetter J. (2008). Differential effects of cannabinoid receptor agonists on regional brain activity using pharmacological MRI. Br. J. Pharmacol..

[107] Li Y., Kim J. (2015). Neuronal Expression of CB2 Cannabinoid Receptor MRNAs in the Mouse Hippocampus. Neuroscience.

[108] Vaseghi S., Nasehi M., Zarrindast M.R. (2021). How Do Stupendous Cannabinoids Modulate Memory Processing via Affecting Neurotransmitter Systems?. Neurosci. Biobehav. Rev..

[109] Zhang H.Y., Gao M., Liu Q.R., Bi G.H., Li X., Yang H.J., Gardner E.L., Wu J., Xi Z.X. (2014). Cannabinoid CB2 Receptors Modulate Midbrain Dopamine Neuronal Activity and Dopamine-Related Behavior in Mice. Proc. Natl. Acad. Sci. USA.

[110] Eljaschewitsch E., Witting A., Mawrin C., Lee T., Schmidt P.M., Wolf S., Hoertnagl H., Raine C.S., Schneider-Stock R., Nitsch R. (2006). The Endocannabinoid Anandamide Protects Neurons during CNS Inflammation by Induction of MKP-1 in Microglial Cells. Neuron.

[111] Navarro G., Morales P., Rodríguez-Cueto C. (2016). Targeting Cannabinoid CB2 Receptors in the Central Nervous System. Medicinal Chemistry Approaches with Focus on Neurodegenerative Disorders. Front. Neurosci..

[112] Malek N., Popiolek-Barczyk K., Mika J., Przewlocka B., Starowicz K. (2015). Anandamide, Acting via CB2 Receptors, Alleviates LPS-Induced Neuroinflammation in Rat Primary Microglial Cultures. Neural Plast..

[113] Aghazadeh Tabrizi M., Baraldi P.G., Borea P.A., Varani K. (2016). Medicinal Chemistry, Pharmacology, and Potential Therapeutic Benefits of Cannabinoid CB2Receptor Agonists. Chem. Rev..

[114] Reiner A., Heldt S.A., Presley C.S., Guley N.H., Elberger A.J., Deng Y., D’Surney L., Rogers J.T., Ferrell J., Bu W. (2015). Motor, Visual and Emotional Deficits in Mice after Closed-Head Mild Traumatic Brain Injury Are Alleviated by the Novel CB2 Inverse Agonist SMM-189. Int. Mol. J. Sci..

[115] Presley C.S., Mustafa S.M., Abidi A.H., Moore B.M. (2015). Synthesis and biological evaluation of (3′,5′-dichloro-2,6-dihydroxy-biphenyl-4-yl)-aryl/alkyl-methanone selective CB2 inverse agonist. Bioorg. Med. Chem..

[116] Chicca A., Gachet M.S., Petrucci V., Schuehly W., Charles R. (2015). 4′-O-methylhonokiol increases levels of 2-arachidonoyl glycerol in mouse brain via selective inhibition of its COX-2-mediated oxygenation. J. Neuroinflamm..

[117] Nimczick M., Decker M. (2015). New Approaches in the Design and Development of Cannabinoid Receptor Ligands: Multifunctional and Bivalent Compounds. ChemMedChem.

[118] Nimczick M., Pemp D., Darras F.H., Chen X., Heilmann J., Decker M. (2014). Synthesis and biological evaluation of bivalent cannabinoid receptor ligands based on hCB2R selective benzimidazoles reveal unexpected intrinsic properties. Bioorg. Med. Chem..

[119] Khajehali E., Malone D.T., Glass M., Sexton P.M., Christopoulos A., Leach K. (2015). Biased Agonism and Biased Allosteric Modulation at the CB 1 Cannabinoid Receptors. Mol. Pharmacol..

[120] Dhopeshwarkar A., Mackie K. (2014). CB2 cannabinoid receptors as a therapeutic target-what does the future hold?. Mol. Pharmacol..

[121] Morales P., Goya P., Jagerovic N., Hernandez-Folgado L. (2016). Allosteric Modulators of the CB1 Cannabinoid Receptor: A Structural Update Review. Cannabis Cannabionid Res..

[122] Saxena S., Caroni P. (2011). Selective Neuronal Vulnerability in Neurodegenerative Diseases: From Stressor Thresholds to Degeneration. Neuron.

[123] Centonze D., Finazzi-Agrò A., Bernardi G., Maccarrone M. (2007). The Endocannabinoid System in Targeting Inflammatory Neurodegenerative Diseases. Trends Pharmacol. Sci..

[124] Kovacs G.G., Adle-Biassette H., Milenkovic I., Cipriani S., Van Scheppingen J., Aronica E. (2014). Linking pathways in the developing and aging brain with neurodegeneration. Neuroscience.

[125] Di Marzo V., Stella N., Zimmer A. (2015). Endocannabinoid signalling and the deteriorating brain. Nat. Rev. Neurosci..

[126] Scotter E.L., Abood M.E., Glass M. (2010). The Endocannabinoid System as a Target for the Treatment of Neurodegenerative Disease. Br. J. Pharmacol..

[127] Tao R., Li C., Jaffe A.E., Shin J.H., Deep-Soboslay A., Yamin R., Weinberger D.R., Hyde T.M., Kleinman J.E. (2020). Cannabinoid receptor CNR1 expression and DNA methylation in human prefrontal cortex, hippocampus and caudate in brain development and schizophrenia. Transl. Psychiatry.

[128] Monteleone P., Bifulco M., Maina G., Tortorella A., Gazzerro P., Proto M.C., Di Filippo C., Monteleone F., Canestrelli B., Buonerba G. (2010). Investigation of CNR1 and FAAH endocannabinoid gene polymorphisms in bipolar disorder and major depression. Pharmacol. Res..

[129] Demers C.H., Drabant Conley E., Bogdan R., Hariri A.R. (2016). Interactions Between Anandamide and Corticotropin-Releasing Factor Signaling Modulate Human Amygdala Function and Risk for Anxiety Disorders: An Imaging Genetics Strategy for Modeling Molecular Interactions. Biol. Psychiatry.

[130] Nguyen T., Thomas B.F., Zhang Y. (2019). Overcoming the Psychiatric Side Effects of the Cannabinoid CB1 Receptor Antagonists: Current Approaches for Therapeutics Development. Curr. Top. Med. Chem..

[131] Moreira F.A., Grieb M., Lutz B. (2009). Central side-effects of therapies based on CB1 cannabinoid receptor agonists and antagonists: Focus on anxiety and depression. Best Pract. Res. Clin. Endocrinol. Metab..

[132] Emadi L., Jonaidi H., Amir Abad E.H. (2011). The role of central CB2 cannabinoid receptors on food intake in neonatal chicks. J. Comp. Physiol. A Neuroethol. Sens. Neural Behav. Physiol..

[133] Liu Q.R., Canseco-Alba A., Zhang H.Y., Tagliaferro P., Chung M., Dennis E., Sanabria B., Schanz N., Escosteguy-Neto J.C., Ishiguro H. (2017). Cannabinoid type 2 receptors in dopamine neurons inhibits psychomotor behaviors, alters anxiety, depression and alcohol preference. Sci. Rep..

[134] García-Gutiérrez M.S., Manzanares J. (2011). Overexpression of CB2 cannabinoid receptors decreased vulnerability to anxiety and impaired anxiolytic action of alprazolam in mice. J. Psychopharmacol..

[135] Geresu B., Canseco-Alba A., Sanabria B., Lin Z., Liu Q.R., Onaivi E.S. (2019). Involvement of CB2 receptors in the neurobehavioral effects of *Catha edulis* (Vahl) Endl. (khat) in mice. Molecules.

[136] Svíženská I.H., Brázda V., Klusáková I., Dubový P. (2013). Bilateral Changes of Cannabinoid Receptor Type 2 Protein and mRNA in the Dorsal Root Ganglia of a Rat Neuropathic Pain Model. J. Histochem. Cytochem..

[137] Yu S.J., Reiner D., Shen H., Wu K.J., Liu Q.R., Wang Y. (2015). Time-dependent protection of CB2 receptor agonist in stroke. PLoS ONE.

[138] Ishiguro H., Horiuchi Y., Ishikawa M., Koga M., Imai K., Suzuki Y., Morikawa M., Inada T., Watanabe Y., Takahashi M. (2010). Brain Cannabinoid CB2 Receptor in Schizophrenia. Biol. Psychiatry.

[139] Ortega-Alvaro A., Aracil-Fernández A., García-Gutiérrez M.S., Navarrete F., Manzanares J. (2011). Deletion of CB2 Cannabinoid Receptor Induces Schizophrenia-Related Behaviors in Mice. Neuropsychopharmacology.

[140] Lopez-Rodriguez A.B., Acaz-Fonseca E., Viveros M.P., Garcia-Segura L.M. (2015). Changes in cannabinoid receptors, aquaporin 4 and vimentin expression after traumatic brain injury in adolescent male mice. Association with edema and neurological deficit. PLoS ONE.

[141] Romero-Sandoval E.A., Horvath R., Landry R.P., DeLeo J.A. (2009). Cannabinoid receptor type 2 activation induces a microglial anti-inflammatory phenotype and reduces migration via MKP induction and ERK dephosphorylation. Mol. Pain.

[142] Agudelo M., Yndart A., Morrison M., Figueroa G., Muñoz K., Samikkannu T., Nair M.P. (2013). Differential Expression and Functional Role of Cannabinoid Genes in Alcohol Users. Drug Alcohol Depend..

[143] Geresu B., Engidawork E. (2012). *Catha edulis* F. (Khat) Reverses Haloperidol but Not Morphine Induced Motor Deficits Following Acute and Subacute Administration in Mice. Ethiop. Pharm. J..

[144] Concannon R.M., Okine B.N., Finn D.P., Dowd E. (2015). Differential Upregulation of the Cannabinoid CB2 Receptor in Neurotoxic and Inflammation-Driven Rat Models of Parkinson’s Disease. Exp. Neurol..

[145] Solas M., Francis P.T., Franco R., Ramirez M.J. (2013). CB2 Receptor and Amyloid Pathology in Frontal Cortex of Alzheimer’s Disease Patients. Neurobiol. Aging.

[146] Facchinetti F., Del Giudice E., Furegato S., Passarotto M., Leon A. (2003). Cannabinoids ablate release of TNFα in rat microglial cells stimulated with lypopolysaccharide. Glia.

[147] Lin L., Yihao T., Zhou F., Yin N., Qiang T., Haowen Z., Qianwei C., Jun T., Yuan Z., Gang Z. (2017). Inflammatory regulation by driving microglial M2 polarization: Neuroprotective effects of cannabinoid receptor-2 activation in intracerebral hemorrhage. Front. Immunol..

[148] Ishiguro H., Kibret B.G., Horiuchi Y., Onaivi E.S. (2022). Potential Role of Cannabinoid Type 2 Receptors in Neuropsychiatric and Neurodegenerative Disorders. Front. Psychiatry.

[149] McIntosh T.K., Smith D.H., Meaney D.F., Kotapka M.J., Gennarelli T.A., Graham D.I. (1996). Neuropathological Sequelae of Traumatic Brain Injury: Relationship to Neurochemical and Biomechanical Mechanisms. Lab. Investig..

[150] Block M.L., Hong J.S. (2005). Microglia and Inflammation-Mediated Neurodegeneration: Multiple Triggers with a Common Mechanism. Prog. Neurobiol..

[151] Kreutzberg G.W. (1996). Microglia: A Sensor for Pathological Events in the CNS. Trends Neurosci..

[152] Kurkowska-Jastrzȩbska I., Wrońska A., Kohutnicka M., Członkowski A., Członkowska A. (1999). MHC Class II Positive Microglia and Lymphocytic Infiltration Are Present in the Substantia Nigra and Striatum in Mouse Model of Parkinson’s Disease. Acta Neurobiol. Exp..

[153] Vasincu A., Rusu R.N., Ababei D.C., Larion M., Bild W., Stanciu G.D., Solcan C., Bild V. (2022). Endocannabinoid Modulation in Neurodegenerative Diseases: In Pursuit of Certainty. Biology.

[154] Götz J., Ittner L.M., Schonrock N. (2006). Alzheimer’s Disease and Frontotemporal Dementia: Prospects of a Tailored Therapy?. Med. J. Aust..

[155] Spillantini M.G., Crowther R.A., Jakes R., Hasegawa M., Goedert M. (1998). α-Synuclein in Filamentous Inclusions of Lewy Bodies from Parkinson’s Disease and Dementia with Lewy Bodies. Proc. Natl. Acad. Sci. USA.

[156] Benkler C., O’Neil A.L., Slepian S., Qian F., Weinreb P.H., Rubin L.L. (2018). Aggregated SOD1 Causes Selective Death of Cultured Human Motor Neurons. Sci. Rep..

[157] Hoffner G., Souès S., Djian P. (2007). Aggregation of Expanded Huntingtin in the Brains of Patients with Huntington Disease. Prion.

[158] Hickman S., Izzy S., Sen P., Morsett L., El Khoury J. (2018). Microglia in Neurodegeneration. Nat. Neurosci..

[159] Panatier A., Robitaille R. (2012). The Soothing Touch: Microglial Contact Influences Neuronal Excitability. Dev. Cell.

[160] Nimmerjahn A., Kirchhoff F., Helmchen F. (2005). Neuroscience: Resting Microglial Cells Are Highly Dynamic Surveillants of Brain Parenchyma In Vivo. Science.

[161] Paolicelli R.C., Bolasco G., Pagani F., Maggi L., Scianni M., Panzanelli P., Giustetto M., Ferreira T.A., Guiducci E., Dumas L. (2011). Synaptic Pruning by Microglia Is Necessary for Normal Brain Development. Science.

[162] Lan X., Han X., Li Q., Yang Q.W., Wang J. (2017). Modulators of microglial activation and polarization after intracerebral haemorrhage. Nat. Rev. Neurol..

[163] Sierra A., Abiega O., Shahraz A., Neumann H. (2013). Janus-Faced Microglia: Beneficial and Detrimental Consequences of Microglial Phagocytosis. Front. Cell. Neurosci..

[164] Helmut K., Hanisch U.K., Noda M., Verkhratsky A. (2011). Physiology of Microglia. Physiol. Rev..

[165] Tang Y., Le W. (2016). Differential Roles of M1 and M2 Microglia in Neurodegenerative Diseases. Mol. Neurobiol..

[166] Naguib M., Xu J.J., Diaz P., Brown D.L., Cogdell D., Bie B., Hu J., Craig S., Hittelman W.N. (2012). Prevention of paclitaxel-induced neuropathy through activation of the central cannabinoid type 2 receptor system. Anesth. Analg..

[167] Tsuda M., Shigemoto-Mogami Y., Koizumi S., Mizokoshi A., Kohsaka S., Salter M.W., Inoue K. (2003). P2X4 receptors induced in spinal microglia gate tactile allodynia after nerve injury. Nature.

[168] Cassano T., Calcagnini S., Pace L., Marco F., De Romano A., Gaetani S. (2017). Cannabinoid receptor 2 signaling in neurodegenerative disorders: From pathogenesis to a promising therapeutic target. Front. Neurosci..

[169] Mecha M., Carrillo-Salinas F.J., Feliú A., Mestre L., Guaza C. (2016). Microglia Activation States and Cannabinoid System: Therapeutic Implications. Pharmacol. Ther..

[170] Turcotte C., Blanchet M.R., Laviolette M., Flamand N. (2016). The CB2 Receptor and Its Role as a Regulator of Inflammation. Cell. Mol. Life Sci..

[171] Correa F., Hernangómez M., Mestre L., Loría F., Spagnolo A., Docagne F., Di Marzo V., Guaza C. (2010). Anandamide Enhances IL-10 Production in Activated Microglia by Targeting CB2 Receptors: Roles of ERK1/2, JNK, and NF-ΚB. Glia.

[172] Oddi S., Latini L., Viscomi M.T., Bisicchia E., Molinari M., Maccarrone M. (2012). Distinct Regulation of NNOS and INOS by CB2 Receptor in Remote Delayed Neurodegeneration. J. Mol. Med..

[173] Viscomi M.T., Oddi S., Latini L., Pasquariello N., Florenzano F., Bernardi G., Molinari M., Maccarrone M. (2009). Selective CB2 Receptor Agonism Protects Central Neurons from Remote Axotomy-Induced Apoptosis through the PI3K/Akt Pathway. J. Neurosci..

[174] Molina-Holgado F., Pinteaux E., Moore J.D., Molina-Holgado E., Guaza C., Gibson R.M., Rothwell N.J. (2003). Endogenous Interleukin-1 Receptor Antagonist Mediates Anti-Inflammatory and Neuroprotective Actions of Cannabinoids in Neurons and Glia. J. Neurosci..

[175] Smith S.R., Terminelli C., Denhardt G. (2000). Effects of Cannabinoid Receptor Agonist and Antagonist Ligands on Production of Inflammatory Cytokines and Anti-Inflammatory Interleukin-10 in Endotoxemic Mice. J. Pharmacol. Exp. Ther..

[176] Amenta P.S., Jallo J.I., Tuma R.F., Elliott M.B. (2012). A Cannabinoid Type 2 Receptor Agonist Attenuates Blood-Brain Barrier Damage and Neurodegeneration in a Murine Model of Traumatic Brain Injury. J. Neurosci. Res..

[177] Bachiller S., Jiménez-Ferrer I., Paulus A., Yang Y., Swanberg M., Deierborg T., Boza-Serrano A. (2018). Microglia in Neurological Diseases: A Road Map to Brain-Disease Dependent-Inflammatory Response. Front. Cell. Neurosci..

[178] Sánchez A.J., García-Merino A. (2012). Neuroprotective agents: Cannabinoids. Clin. Immunol..

[179] Fernández-Ruiz J., Pazos M.R., García-Arencibia M., Sagredo O., Ramos J.A. (2008). Role of CB2 receptors in neuroprotective effects of cannabinoids. Mol. Cell. Endocrinol..

[180] Gómez-Gálvez Y., Palomo-Garo C., Fernández-Ruiz J., García C. (2016). Potential of the cannabinoid CB2 receptor as a pharmacological target against inflammation in Parkinson’s disease. Prog. Neuropsychopharmacol. Biol. Psychiatry.

[181] Xin Q., Xu F., Taylor D.H., Zhao J.F., Wu J. (2020). The impact of cannabinoid type 2 receptors (CB2Rs) in neuroprotection against neurological disorders. Acta Pharmacol. Sin..

[182] GBD 2019 Dementia Forecasting Collaborators (2022). Estimation of the global prevalence of dementia in 2019 and forecasted prevalence in 2050: An analysis for the Global Burden of Disease Study 2019. Lancet. Public Health.

[183] Condello C., Yuan P., Schain A., Grutzendler J. (2015). Microglia constitute a barrier that prevents neurotoxic protofibrillar Aβ42 hotspots around plaques. Nat. Commun..

[184] Perea J.R., Llorens-Martín M., Ávila J., Bolós M. (2018). The Role of Microglia in the Spread of Tau: Relevance for Tauopathies. Front. Cell. Neurosci..

[185] Heneka M.T., O’Banion M.K., Terwel D., Kummer M.P. (2010). Neuroinflammatory processes in Alzheimer’s disease. J. Neural Transm..

[186] Walter L., Franklin A., Witting A., Wade C., Xie Y., Kunos G., Mackie K., Stella N. (2003). Nonpsychotropic cannabinoid receptors regulate microglial cell migration. J. Neurosci..

[187] Benito C., Núñez E., Tolón R.M., Carrier E.J., Rábano A., Hillard C.J., Romero J. (2003). Cannabinoid CB2 receptors and fatty acid amide hydrolase are selectively overexpressed in neuritic plaque-associated glia in Alzheimer’s disease brains. J. Neurosci..

[188] Ramírez B.G., Blázquez C., Gómez del Pulgar T., Guzmán M., de Ceballos M.L. (2005). Prevention of Alzheimer’s disease pathology by cannabinoids: Neuroprotection mediated by blockade of microglial activation. J. Neurosci..

[189] Wang L., Liu B.-J., Cao Y., Xu W.-Q., Sun D.-S., Li M.-Z., Shi F.-X., Li M., Tian Q., Wang J.Z. (2018). Deletion of Type-2 Cannabinoid Receptor Induces Alzheimer’s Disease-like Tau Pathology and Memory Impairment through AMPK/GSK3β Pathway. Mol. Neurobiol..

[190] Koppel J., Vingtdeux V., Marambaud P., d’Abramo C., Jimenez H., Stauber M., Friedman R., Davies P. (2014). CB2 Receptor Deficiency Increases Amyloid Pathology and Alters Tau Processing in a Transgenic Mouse Model of Alzheimer’s Disease. Mol. Med..

[191] Tolón R.M., Núñez E., Pazos M.R., Benito C., Castillo A.I., Martínez-Orgado J.A., Romero J. (2009). The activation of cannabinoid CB2 receptors stimulates in situ and in vitro beta-amyloid removal by human macrophages. Brain Res..

[192] Li C., Shi J., Wang B., Li J., Jia H. (2019). CB2 cannabinoid receptor agonist ameliorates novel object recognition but not spatial memory in transgenic APP/PS1 mice. Neurosci. Lett..

[193] Aso E., Juvés S., Maldonado R., Ferrer I. (2013). CB2 cannabinoid receptor agonist ameliorates Alzheimer-like phenotype in AβPP/PS1 mice. J. Alzheimer’s Dis..

[194] Martín-Moreno A.M., Brera B., Spuch C., Carro E., García-García L., Delgado M., Pozo M.A., Innamorato N.G., Cuadrado A., de Ceballos M.L. (2012). Prolonged oral cannabinoid administration prevents neuroinflammation, lowers β-amyloid levels and improves cognitive performance in Tg APP 2576 mice. J. Neuroinflamm..

[195] Wu J., Hocevar M., Foss J.F., Bie B., Naguib M. (2017). Activation of CB (2) receptor system restores cognitive capacity and hippocampal Sox2 expression in a transgenic mouse model of Alzheimer’s disease. Eur. J. Pharmacol..

[196] Wu J., Bie B., Yang H., Xu J.J., Brown D.L., Naguib M. (2013). Activation of the CB2 receptor system reverses amyloid-induced memory deficiency. Neurobiol. Aging.

[197] Schmöle A.-C., Lundt R., Toporowski G., Hansen J.N., Beins E., Halle A., Zimmer A. (2018). Cannabinoid Receptor 2-Deficiency Ameliorates Disease Symptoms in a Mouse Model with Alzheimer’s Disease-like Pathology. J. Alzheimer’s Dis..

[198] Galán-Ganga M., Rodríguez-Cueto C., Merchán-Rubira J., Hernández F., Ávila J., Posada-Ayala M., Lanciego J.L., Luengo E., Lopez M.G., Rábano A. (2021). Cannabinoid receptor CB2 ablation protects against TAU induced neurodegeneration. Acta Neuropathol. Commun..

[199] Baul H.S., Manikandan C., Sen D. (2018). Cannabinoid receptor as a potential therapeutic target for Parkinson’s Disease. Brain Res. Bull..

[200] Han Q.W., Yuan Y.H., Chen N.H. (2019). The therapeutic role of cannabinoid receptors and its agonists or antagonists in Parkinson’s disease. Prog. Neuropsychopharmacol. Biol. Psychiatry.

[201] Kelly R., Joers V., Tansey M.G., McKernan D.P., Dowd E. (2020). Microglial phenotypes and their relationship to the cannabinoid system: Therapeutic implications for Parkinson’s disease. Molecules.

[202] De Lago E., Fernandez-Ruiz J. (2007). Cannabinoids and neuroprotection in motor-related disorders. CNS Neurol. Disord. Drug Targets.

[203] Javed H., Azimullah S., Haque M.E., Ojha S.K. (2016). Cannabinoid type 2 (CB2) receptors activation protects against oxidative stress and neuroinflammation associated dopaminergic neurodegeneration in rotenone model of Parkinson’s disease. Front. Neurosci..

[204] García M.C., Cinquina V., Palomo-Garo C., Rábano A., Fernández-Ruiz J. (2015). Identification of CB2 receptors in human nigral neurons that degenerate in Parkinson’s disease. Neurosci. Lett..

[205] Navarrete F., García-Gutiérrez M.S., Aracil-Fernández A., Lanciego J.L., Manzanares J. (2018). Cannabinoid cb1 and cb2 receptors, and monoacylglycerol lipase gene expression alterations in the basal ganglia of patients with Parkinson’s disease. Neurotherapeutics.

[206] The Huntington’s Disease Collaborative Research Group (1993). A novel gene containing a trinucleotide repeat that is expanded and unstable on Huntington’s disease chromosomes. The Huntington’s Disease Collaborative Research Group. Cell.

[207] Myers R.H. (2004). Huntington’s disease genetics. NeuroRx.

[208] Chen M., Wolynes P.G. (2017). Aggregation landscapes of Huntingtin exon 1 protein fragments and the critical repeat length for the onset of Huntington’s disease. Proc. Natl. Acad. Sci. USA.

[209] Duyao M., Ambrose C., Myers R., Novelletto A., Persichetti F., Frontali M., Folstein S., Ross C., Franz M., Abbott M. (1993). Trinucleotide repeat length instability and age of onset in Huntington’s disease. Nat. Genet..

[210] Pringsheim T., Wiltshire K., Day L., Dykeman J., Steeves T., Jette N. (2012). The incidence and prevalence of Huntington’s disease: A systematic review and meta-analysis. Mov. Disord..

[211] Ross C.A., Aylward E.H., Wild E.J., Langbehn D.R., Long J.D., Warner J.H., Scahill R.I., Leavitt B.R., Stout J.C., Paulsen J.S. (2014). Huntington disease: Natural history, biomarkers and prospects for therapeutics. Nat. Rev. Neurol..

[212] Yang H.M., Yang S., Huang S.-S., Tang B.-S., Guo J.F. (2017). Microglial Activation in the Pathogenesis of Huntington’s Disease. Front. Aging Neurosci..

[213] Tai Y.F., Pavese N., Gerhard A., Tabrizi S.J., Barker R.A., Brooks D.J., Piccini P. (2007). Imaging microglial activation in Huntington’s disease. Brain Res. Bull..

[214] Björkqvist M., Wild E.J., Thiele J., Silvestroni A., Andre R., Lahiri N., Raibon E., Lee R.V., Benn C.L., Soulet D. (2008). A novel pathogenic pathway of immune activation detectable before clinical onset in Huntington’s disease. J. Exp. Med..

[215] Silvestroni A., Faull R.L.M., Strand A.D., Möller T. (2009). Distinct neuroinflammatory profile in post-mortem human Huntington’s disease. Neuroreport.

[216] Politis M., Lahiri N., Niccolini F., Su P., Wu K., Giannetti P., Scahill R.I., Turkheimer F.E., Tabrizi S.J., Piccini P. (2015). Increased central microglial activation associated with peripheral cytokine levels in premanifest Huntington’s disease gene carriers. Neurobiol. Dis..

[217] Chang R., Liu X., Li S., Li X.-J. (2015). Transgenic animal models for study of the pathogenesis of Huntington’s disease and therapy. Drug Des. Dev. Ther..

[218] Lu B., Palacino J. (2013). A novel human embryonic stem cell-derived Huntington’s disease neuronal model exhibits mutant huntingtin (mHTT) aggregates and soluble mHTT-dependent neurodegeneration. FASEB J..

[219] Xu X., Tay Y., Sim B., Yoon S.-I., Huang Y., Ooi J., Utami K.H., Ziaei A., Ng B., Radulescu C. (2017). Reversal of Phenotypic Abnormalities by CRISPR/Cas9-Mediated Gene Correction in Huntington Disease Patient-Derived Induced Pluripotent Stem Cells. Stem Cell Rep..

[220] Ferrante R.J. (2009). Mouse models of Huntington’s disease and methodological considerations for therapeutic trials. Biochim. Biophys. Acta—Mol. Basis Dis..

[221] Denovan-Wright E.M., Robertson H.A. (2000). Cannabinoid receptor messenger RNA levels decrease in a subset of neurons of the lateral striatum, cortex and hippocampus of transgenic Huntington’s disease mice. Neuroscience.

[222] Glass M., Dragunow M., Faull R.L.M. (2000). The pattern of neurodegeneration in Huntington’s disease: A comparative study of cannabinoid, dopamine, adenosine and GABA(A) receptor alterations in the human basal ganglia in Huntington’s disease. Neuroscience.

[223] Van Laere K., Casteels C., Dhollander I., Goffin K., Grachev I., Bormans G., Vandenberghe W. (2010). Widespread decrease of type 1 cannabinoid receptor availability in Huntington disease in vivo. J. Nucl. Med..

[224] Bouchard J., Truong J., Bouchard K., Dunkelberger D., Desrayaud S., Moussaoui S., Tabrizi S.J., Stella N., Muchowski P.J. (2012). Cannabinoid receptor 2 signaling in peripheral immune cells modulates disease onset and severity in mouse models of Huntington’s disease. J. Neurosci..

[225] Palazuelos J., Aguado T., Pazos M.R., Julien B., Carrasco C., Resel E., Sagredo O., Benito C., Romero J., Azcoitia I. (2009). Microglial CB2 cannabinoid receptors are neuroprotective in Huntington’s disease excitotoxicity. Brain.

[226] Sagredo O., González S., Aroyo I., Pazos M.R., Benito C., Lastres-Becker I., Romero J.P., Tolón R.M., Mechoulam R., Brouillet E. (2009). Cannabinoid CB2 receptor agonists protect the striatum against malonate toxicity: Relevance for Huntington’s disease. Glia.

[227] Dowie M.J., Grimsey N.L., Hoffman T., Faull R.L.M., Glass M. (2014). Cannabinoid receptor CB2 is expressed on vascular cells, but not astroglial cells in the post-mortem human Huntington’s disease brain. J. Chem. Neuroanat..

[228] Jankovska N., Matej R. (2021). Molecular Pathology of ALS: What We Currently Know and What Important Information Is Still Missing. Diagnostics.

[229] Ragagnin A., Shadfar S., Vidal M., Jamali M.S., Atkin J.D. (2019). Motor Neuron Susceptibility in ALS/FTD. Front. Neurosci..

[230] Motataianu A., Serban G., Barcutean L., Balasa R. (2022). Oxidative Stress in Amyotrophic Lateral Sclerosis: Synergy of Genetic and Environmental Factors. Int. J. Mol. Sci..

[231] Hampson A.J., Grimaldi M. (2001). Cannabinoid receptor activation and elevated cyclic AMP reduce glutamate neurotoxicity. Eur. J. Neurosci..

[232] Musetti B., González-Ramos H., González M., Bahnson E.M., Varela J., Thomson L. (2020). Cannabis sativa extracts protect LDL from Cu^2+^-mediated oxidation. J. Cannabis Res..

[233] Borges R.S., Batista J., Viana R.B., Baetas A.C., Orestes E., Andrade M.A., Honório K.M., Da Silva A.B.F. (2013). Understanding the Molecular Aspects of Tetrahydrocannabinol and Cannabidiol as Antioxidants. Molecules.

[234] Atalay S., Jarocka-Karpowicz I., Skrzydlewska E. (2020). Antioxidative and Anti-Inflammatory Properties of Cannabidiol. Antioxidants.

[235] Yiangou Y., Facer P., Durrenberger P., Chessell I.P., Naylor A., Bountra C., Banati R.R., Anand P. (2006). COX-2, CB2 and P2X7-immunoreactivities are increased in activated microglial cells/macrophages of multiple sclerosis and amyotrophic lateral sclerosis spinal cord. BMC Neurol..

[236] Espejo-Porras F., Fernández-Ruiz J., de Lago E. (2018). Analysis of endocannabinoid receptors and enzymes in the post-mortem motor cortex and spinal cord of amyotrophic lateral sclerosis patients. Amyotroph. Lateral Scler. Frontotempor. Degener..

[237] Shoemaker J.L., Seely K.A., Reed R.L., Crow J.P., Prather P.L. (2007). The CB2 cannabinoid agonist AM-1241 prolongs survival in a transgenic mouse model of amyotrophic lateral sclerosis when initiated at symptom onset. J. Neurochem..

[238] Moreno-Martet M., Espejo-Porras F., Fernández-Ruiz J., de Lago E. (2014). Changes in endocannabinoid receptors and enzymes in the spinal cord of SOD1(G93A) transgenic mice and evaluation of a Sativex(^®^)-like combination of phytocannabinoids: Interest for future therapies in amyotrophic lateral sclerosis. CNS Neurosci. Ther..

[239] Fernández-Trapero M., Espejo-Porras F., Rodríguez-Cueto C., Coates J.R., Pérez-Díaz C., de Lago E., Fernández-Ruiz J. (2017). Upregulation of CB2 receptors in reactive astrocytes in canine degenerative myelopathy, a disease model of amyotrophic lateral sclerosis. Dis. Models Mech..

[240] Espejo-Porras F., Piscitelli F., Verde R., Ramos J.A., Di Marzo V., de Lago E., Fernández-Ruiz J. (2015). Changes in the endocannabinoid signaling system in CNS structures of TDP-43 transgenic mice: Relevance for a neuroprotective therapy in TDP-43-related disorders. J. Neuroimmune Pharmacol..

[241] Witting A., Weydt P., Hong S., Kliot M., Moller T., Stella N. (2004). Endocannabinoids accumulate in spinal cord of SOD1 G93A transgenic mice. J. Neurochem..

[242] Bilsland L.G., Dick J.R., Pryce G., Petrosino S., Di Marzo V., Baker D., Greensmith L. (2006). Increasing cannabinoid levels by pharmacological and genetic manipulation delay disease progression in SOD1 mice. FASEB J..

[243] Kim K., Moore D.H., Makriyannis A., Abood M.E. (2006). AM1241, a cannabinoid CB2 receptor selective compound, delays disease progression in a mouse model of amyotrophic lateral sclerosis. Eur. J. Pharmacol..

[244] Weydt P., Hong S., Witting A., Möller T., Stella N., Kliot M. (2005). Cannabinol delays symptom onset in SOD1 (G93A) transgenic mice without affecting survival. Amyotroph. Lateral Scler. Other Motor Neuron Disord..

[245] Espejo-Porras F., García-Toscano L., Rodríguez-Cueto C., Santos-García I., de Lago E., Fernandez-Ruiz J. (2019). Targeting glial cannabinoid CB (2) receptors to delay the progression of the pathological phenotype in TDP-43 (A315T) transgenic mice, a model of amyotrophic lateral sclerosis. Br. J. Pharmacol..

[246] Rodríguez-Cueto C., Gómez-Almería M., García Toscano L., Romero J., Hillard C.J., de Lago E., Fernández-Ruiz J. (2021). Inactivation of the CB2 receptor accelerated the neuropathological deterioration in TDP-43 transgenic mice, a model of amyotrophic lateral sclerosis. Brain Pathol..

[247] Amtmann D., Weydt P., Johnson K.L., Jensen M.P., Carter G.T. (2004). Survey of cannabis use in patients with amyotrophic lateral sclerosis. Am. J. Hosp. Paliat. Care.

[248] Urbi B., Owusu M.A., Hughes I., Katz M., Broadley S., Sabet A. (2019). Effects of cannabinoids in Amyotrophic Lateral Sclerosis (ALS) murine models: A systematic review and meta-analysis. J. Neurochem..

[249] Yang J.H., Rempe T., Whitmire N., Dunn-Pirio A., Graves J.S. (2022). Therapeutic Advances in Multiple Sclerosis. Front. Neurol..

[250] Wagner C.A., Roqué P.J., Goverman J.M. (2020). Pathogenic T cell cytokines in multiple sclerosis. J. Exp. Med..

[251] Palazuelos J., Davoust N., Julien B., Hatterer E., Aguado T., Mechoulam R., Benito C., Romero J., Silva A., Guzmán M. (2008). The CB (2) cannabinoid receptor controls myeloid progenitor trafficking: Involvement in the pathogenesis of an animal model of multiple sclerosis. J. Biol. Chem..

[252] Kong W., Li H., Tuma R.F., Ganea D. (2014). Selective CB2 receptor activation ameliorates EAE by reducing Th17 differentiation and immune cell accumulation in the CNS. Cell. Immunol..

[253] Maresz K., Pryce G., Ponomarev E.D., Marsicano G., Croxford J.L., Shriver L.P., Ledent C., Cheng X., Carrier E.J., Mann M.K. (2007). Direct suppression of CNS autoimmune inflammation via the cannabinoid receptor CB1 on neurons and CB2 on autoreactive T cells. Nat. Med..

[254] Zhang M., Martin B.R., Adler M.W., Razdan R.J., Kong W., Ganea D., Tuma R.F. (2009). Modulation of cannabinoid receptor activation as a neuroprotective strategy for EAE and stroke. J. Neuroimmune Pharmacol..

[255] Rossi S., Furlan R., De Chiara V., Muzio L., Musella A., Motta C., Studer V., Cavasinni F., Bernardi G., Martino G. (2011). Cannabinoid CB1 receptors regulate neuronal TNF-α effects in experimental autoimmune encephalomyelitis. Brain Behav. Immun..

[256] Cencioni M.T., Chiurchiù V., Catanzaro G., Borsellino G., Bernardi G., Battistini L., Maccarrone M. (2010). Anandamide suppresses proliferation and cytokine release from primary human T-lymphocytes mainly via CB2 receptors. PLoS ONE.

[257] Sánchez López A.J., Román-Vega L., Ramil Tojeiro E., Giuffrida A., García-Merino A. (2015). Regulation of cannabinoid receptor gene expression and endocannabinoid levels in lymphocyte subsets by interferon-β: A longitudinal study in multiple sclerosis patients. Clin. Exp. Immunol..

[258] Maresz K., Carrier E.J., Ponomarev E.D., Hillard C.J., Dittel B.N. (2005). Modulation of the cannabinoid CB2 receptor in microglial cells in response to inflammatory stimuli. J. Neurochem..

[259] Tahamtan A., Rezaiy S., Samadizadeh S., Moradi A., Tabarraei A., Javid N., Oladnabi M., Naeimi M.H. (2020). Cannabinoid CB2 Receptor Functional Variation (Q63R) Is Associated with Multiple Sclerosis in Iranian Subjects. J. Mol. Neurosci..

[260] Carrasquer A., Nebane N.M., Williams W.M., Song Z.H. (2010). Functional consequences of nonsynonymous single nucleotide polymorphisms in the CB2 cannabinoid receptor. Pharmacogenet. Genom..

[261] Kozela E., Lev N., Kaushansky N., Eilam R., Rimmerman N., Levy R., Ben-Nun A., Juknat A., Vogel Z. (2011). Cannabidiol inhibits pathogenic T cells, decreases spinal microglial activation and ameliorates multiple sclerosis-like disease in C57BL/6 mice. Br. J. Pharmacol..

[262] Haddad F., Dokmak G., Karaman R. (2022). The Efficacy of Cannabis on Multiple Sclerosis-Related Symptoms. Life.

[263] Longoria V., Parcel H., Toma B., Minhas A., Zeine R. (2022). Neurological Benefits, Clinical Challenges, and Neuropathologic Promise of Medical Marijuana: A Systematic Review of Cannabinoid Effects in Multiple Sclerosis and Experimental Models of Demyelination. Biomedicines.

[264] Arévalo-Martín A., Vela J.M., Molina-Holgado E., Borrell J., Guaza C. (2003). Therapeutic action of cannabinoids in a murine model of multiple sclerosis. J. Neurosci..

[265] Friese M.A., Schattling B., Fugger L. (2014). Mechanisms of neurodegeneration and axonal dysfunction in multiple sclerosis. Nat. Rev. Neurol..

[266] Malfitano A.M., Laezza C., D’Alessandro A., Procaccini C., Saccomanni G., Tuccinardi T., Manera C., Macchia M., Matarese G., Gazzerro P. (2013). Effects on immune cells of a new 1,8-naphthyridin-2-one derivative and its analogues as selective CB2 agonists: Implications in multiple sclerosis. PLoS ONE.

[267] Alberti T.B., Barbosa W.L.R., Vieira J.L.F., Raposo N.R.B., Dutra R.C. (2017). (−)-β-Caryophyllene, a CB2 Receptor-Selective Phytocannabinoid, Suppresses Motor Paralysis and Neuroinflammation in a Murine Model of Multiple Sclerosis. Int. J. Mol. Sci..

[268] Vermersch P. (2011). Sativex(^®^) (tetrahydrocannabinol + cannabidiol), an endocannabinoid system modulator: Basic features and main clinical data. Expert Rev. Neurother..

[269] Notcutt W.G. (2015). Clinical use of cannabinoids for symptom control in multiple sclerosis. Neurotherapeutics.

[270] Fernández-Ruiz J., García C., Sagredo O., Gómez-Ruiz M., de Lago E. (2010). The endocannabinoid system as a target for the treatment of neuronal damage. Expert Opin. Ther. Targets.

[271] Fernández-Ruiz J., Moro M.A., Martínez-Orgado J. (2015). Cannabinoids in Neurodegenerative Disorders and Stroke/Brain Trauma: From Preclinical Models to Clinical Applications. Neurotherapeutics.

[272] Pertwee R.G. (2005). Pharmacological actions of cannabinoids. Cannabinoids.

[273] De Aquino J.P., Sherif M., Radhakrishnan R., Cahill J.D., Ranganathan M., D’Souza D.C. (2018). The Psychiatric Consequences of Cannabinoids. Clin. Ther..

[274] Dhopeshwarkar A., Mackie K. (2016). Functional Selectivity of CB2 Cannabinoid Receptor Ligands at a Canonical and Noncanonical Pathway. J. Pharmacol. Exp. Ther..

[275] Callén L., Moreno E., Barroso-Chinea P., Moreno-Delgado D., Cortés A., Mallol J., Casadó V., Lanciego J.L., Franco R., Lluis C. (2012). Cannabinoid receptors CB1 and CB2 form functional heteromers in brain. J. Biol. Chem..

[276] Shademan B., Biray Avci C., Nikanfar M., Nourazarian A. (2021). Application of Next-Generation Sequencing in Neurodegenerative Diseases: Opportunities and Challenges. Neuromol. Med..

[277] Scimone C., Donato L., Alibrandi S., Esposito T., Alafaci C., D’Angelo R., Sidoti A. (2020). Transcriptome analysis provides new molecular signatures in sporadic Cerebral Cavernous Malformation endothelial cells. Biochim. Biophys. Acta—Mol. Basis Dis..

[278] Scimone C., Donato L., Alafaci C., Granata F., Rinaldi C., Longo M., D’Angelo R., Sidoti A. (2020). High-Throughput Sequencing to Detect Novel Likely Gene-Disrupting Variants in Pathogenesis of Sporadic Brain Arteriovenous Malformations. Front. Genet..

